# Bayesian mixed models for longitudinal genetic data: theory, concepts, and simulation studies

**DOI:** 10.5808/gi.21080

**Published:** 2022-03-31

**Authors:** Wonil Chung, Youngkwang Cho

**Affiliations:** 1 Department of Statistics and Actuarial Science, Soongsil University, Seoul 06978, Korea; 2 Program in Genetic Epidemiology and Statistical Genetics, Harvard T.H. Chan School of Public Health, Boston, MA 02115, USA

**Keywords:** Bayesian mixed model, gene-time interaction, grid-based model, longitudinal data

## Abstract

Despite the success of recent genome-wide association studies investigating longitudinal traits, a large fraction of overall heritability remains unexplained. This suggests that some of the missing heritability may be accounted for by gene-gene and gene-time/environment interactions. In this paper, we develop a Bayesian variable selection method for longitudinal genetic data based on mixed models. The method jointly models the main effects and interactions of all candidate genetic variants and non-genetic factors and has higher statistical power than previous approaches. To account for the within-subject dependence structure, we propose a grid-based approach that models only one fixed-dimensional covariance matrix, which is thus applicable to data where subjects have different numbers of time points. We provide the theoretical basis of our Bayesian method and then illustrate its performance using data from the 1000 Genome Project with various simulation settings. Several simulation studies show that our multivariate method increases the statistical power compared to the corresponding univariate method and can detect gene-time/environment interactions well. We further evaluate our method with different numbers of individuals, variants, and causal variants, as well as different trait-heritability, and conclude that our method performs reasonably well with various simulation settings.

## Introduction

In recent years, genome-wide association studies (GWAS) for longitudinal traits (e.g., body weight or cholesterol levels) have been carried out in cohorts, where multiple measurements have been collected from each individual [1-7]. Although GWAS have successfully discovered a large number of novel genetic variants associated with these traits, the identified variants typically account for only a small proportion of overall heritability [8-10]. A presumed explanation for the “missing heritability” is that existing methods have low power to identify gene-gene and gene-time/environment interactions [11]. Since traditional methodologies are limited to the identification of variants with marginal effects using a single measurement per individual, a large amount of useful information in longitudinal data is lost and variants that interact with other variants or have time-varying effects may not be detected [12]. It is more appropriate to analyze multiple variants simultaneously, using all available measurements, for longitudinal genetic studies.

There are methodological challenges associated with the genetic analysis of longitudinal traits for multiple variants. Most complex traits are typically controlled by multiple variants that interact with each other or environmental factors. It may be exceedingly difficult to model all candidate variants with epistasis effect and gene-time/environment interactions for longitudinal traits because genetic data are generally high-dimensional relative to the number of samples. Bayesian multiple quantitative trait loci (QTL) mapping methods [13-16] have been proposed for modeling epistatic effects. Multiple QTL can be simultaneously detected by treating the number of QTL as a random variable using the reversible jump Markov-chain Monte Carlo (MCMC) method [13,14]. Alternatively, multiple QTL can be viewed as a variable selection problem [15,16]. Bayesian model selection approaches are used for identifying QTL with main and epistatic effects [17], as well as QTL that interact with other covariates [18] based on the composite model space framework. These approaches use a fixed-dimensional parameter space by setting an upper bound on the number of detectable QTL and introduce latent binary variables for deciding which variables will be included in the model. This technique reasonably reduces the model space using efficient MCMC algorithms. For multiple QTL mapping with multivariate traits, Banerjee et al. [19] extended the Bayesian variable selection method of Yi [16] via a model that allows different genetic models for different traits. This method provides a multiple QTL mapping strategy for correlated traits, but it does not account for the dependence structure among repeated measurements from each subject.

Several statistical methods have been proposed for dealing with within-subject variation. For data collected at the same time points across all individuals, the measured values at each time point can be treated as one variable. The data can then be treated as multivariate outcomes and jointly analyzed [20-23]. For data collected at different time points across some or all individuals, the measured values cannot be effectively grouped; thus, standard multivariate analysis is no longer applicable. Alternatively, mixed models are used for longitudinal data to map QTL [24]. Mixed models are flexible in modeling such unbalanced data because they allow non-constant correlations among observations. Chung and Zou [25] developed a Bayesian multiple association-mapping algorithm based on a mixed model with a built-in variable selection feature. It models multiple genes simultaneously and allows gene-gene and gene-time/environment interactions for repeatedly measured phenotypes. However, in that model, we made the strong assumption that the covariance matrix is known up to a constant. We plan to relax that assumption here.

In this paper, we develop a Bayesian variable selection method for longitudinal data where phenotypes are not measured at a fixed set of time points for all samples. It jointly models the main and pairwise interactions of all candidate genetic variants. We propose a novel grid-based approach to parsimoniously model each subject's covariance matrix as a function of a covariance matrix defined on a set of pre-selected time points where each observed time point is mapped to its two adjacent grid time points via linear interpolation. This approach thus deals only with a covariance matrix of a fixed dimension. The covariance matrix is then modeled nonparametrically using the modified Cholesky decomposition of Chen and Dunson [26], which facilitates the use of normal conjugate priors. The deviance information criterion (DIC) and the Bayesian predictive information criterion (BPIC) are proposed for the selection of an optimal number of grid points. The paper is organized as follows. In the Methods section, we introduce a novel grid-based Bayesian method for longitudinal genetic data and provide its theoretical basis. In the Results section, we show numerous simulation results using whole-genome sequencing data from the 1000 Genome Project to evaluate the performance of the proposed methods and assess the effects of sample size, number of variants, causal variants, and heritability. We conclude the paper with some discussions on the proposed methods and future research.

## Methods

### Genotype data

For our simulation studies, we utilized the whole-genome sequencing data from the 1000 Genome Project, which created a catalogue of common human variations using samples from people who provided open consent who declared themselves healthy. It ran between 2008 and 2015, generating a large public catalogue of human variations and genotype data. We randomly selected 400 out of 504 individuals of East Asian (EAS) ancestry from the 1000 Genome Project data (phase 3 version 5) and then removed single-nucleotide polymorphisms (SNPs) with a minor allele frequency <5% and p(Hardy-Weinberg equilibrium) <10^-6^, which resulted in 6, 247, 288 SNPs.

### Bayesian mixed models

For a given trait, suppose we have *n* individuals where individual *i* has phenotypes measured at *n_i_* time points (*i*=1, ..., *n*) and *p* SNPs. Let $N=\sum_{i=1}^{n}n_{i}$. We set the number of main effect terms equal to *p*, the number of SNP-SNP interaction terms to $\frac{p(p-1)}{2}$, and the number of SNP-covariate interaction terms to *pq*, where *q* is the number of covariates in the model, including time. We define *λ*= (*λ*_1_, ..., *λ_d_*)^*T*^ as the SNP positions associated with the above genetic effects, where $d=p+\frac{p(p-1)}{2}+pq$. Each SNP can be associated with the trait through its main effect or interactions with other SNPs (epistatic effects) or covariates. We introduce latent binary variables *γ*=(*γ*_1_, ..., *γ_d_*)^*T*^ for the selection of genetic effects to be included in (*γ_i_*=1) or excluded from (*γ_i_*=0) the model. The vector (*γ*, *λ*) determines the number and positions of SNPs. For the *i*th individual, *x_ti_* denotes the *n_i_*×*q* design matrix of time/environmental covariates, *x_gi_* denotes the *n_i_*×*p* design matrix of the *p* SNPs, *x_ggi_* denotes the $n_{i}\times \frac{p(p-1)}{2}$ design matrix of the epistatic effects, and *x_gti_* denotes the *n_i_*×*pq* design matrix of the SNP-time/SNP-environment interactions. We define the final design matrix as *x_i_*=(*x_ti_*, *x_gi_*, *x_ggi_*, *x_gti_*).

Given *γ*, *λ*, and *x_i_*, we consider the following mixed model:


(1) $$y_{i}=\mu_{i}+x_{i}\Gamma \beta +p_{i}\nu_{i}+e_{i}(i=1,...n),$$

where *y_i_*=(*y*_*i*1_, ..., *y_in_i__*)^*T*^ is an *n_i_*×1 phenotype vector of individual *i*; *μ_i_*=*μ*1_*n_i_*_ is an *n_i_*×1 overall mean vector; *Γ* is a diagonal matrix with upper diagonal elements 1_*q*_ (i.e., the model always contains all non-genetic covariates) and lower diagonal elements $\gamma ;\beta =(\beta_{t}^{T},\;\beta_{g}^{T},\;\beta_{gg}^{T},\;\beta_{gt}^{T}{)}^{T}$ is a vector of genetic effects, time/environmental effects, epistatic effects, and SNP-time/environment interactions; and *e_i_* is an *n_i_*×1 vector of random errors with *e_i_*∼*N*(0, *σ^2^I_n_i__*). To model the correlation among repeated measurements of the same individual, we partition the observed time interval by *k* pre-specified grid points, *t*=(*t*_1_, ..., *t_k_*)^*T*^, and define *ν_i_* as a *k*×1 vector of random effects at the grid time points with *ν_i_*∼*N*(0, *D*) where *D* is a *k*×*k* covariance matrix. Let $p_{i}=(p_{i1}^{T},\;...,\;p_{in_{i}}^{T}{)}^{T}$ and *P*=*diag*(*p*_1_, ..., *p_n_*) where *p_i_* is defined as follows. If all subjects have *k* observations measured exactly on the *k* grid time points, then *p_i_* becomes an identity matrix. We apply an interpolation procedure (e.g., linear, polynomial, or spline) to any observation that does not fall on any of the *k* grid time points. For simplicity, we choose a linear interpolation here. When the *j*th measurement of individual *i* falls at time *t*, which is in between the grid points *t_r_* and *t*_*r*+1_ (*t_r_*≤ *t* ≤ *t*_*r*+1_), we set $p_{ij}=(0_{\left(r-1\right)}^{T},\;\frac{t_{r+1}-t}{t_{r+1}-t_{r}},\;\frac{t-t_{r}}{t_{r+1}-t_{r}},\;0_{\left(k-r-1\right)}^{T})$. When *t*=*t_r_*, we get $p_{ij}=(0_{\left(r-1\right)}^{T},1,\;0_{\left(k-r\right)}^{T})$. We can re-express *p_ij_* as *p_ij_*=*a*_*ij*1_*e*_1_+...+*a_ijk_e_k_*, where *a_ijr_* is the *r*th element of *p_ij_* and *e_r_* (1 ≤ *r* ≤ *k*) is a 1× *k* vector whose elements are all zero except the *r*th component, which equals 1. Note that $\sum_{r=1}^{k}\;a_{ijr}=1,\;0\leq a_{ij1},...,a_{ijk}\leq 1$ and at most two adjacent *a_ijr_* values can be non-zero due to the linear interpolation we employ here.

### Re-parameterized model

For Bayesian estimation of the mixed model (1), we factor *D*, the covariance matrix of the random effects, by employing the modified Cholesky decomposition of Chen and Dunson [26]. Let *L* denote a *k*×*k* lower triangular Cholesky decomposition matrix that has nonnegative diagonal elements, such that *D* = *LL^T^*. Let *L*=*ΔΨ*, where *Δ*=diag(*δ*_1_, ..., *δ_k_*) and *Ψ* is a *k*×*k* matrix with the (*l, m*)th element denoted by *ψ_lm_*. To make *Δ* and *Ψ* identifiable, we make the following assumptions: *δ_l_*≥ 0, *ψ_ll_*=1 and *ψ_lm_*=0, for *l*=1, ..., *k*; *m*=l+1, ..., *k*. These conditions make *Δ* a nonnegative *k*× *k* diagonal matrix and *Ψ* a lower triangular matrix with 1's in the diagonal elements. This leads to the decomposition *D* =*ΔΨΨ^T^Δ*, and thus we reparametrize model (1) as


(2) $$y_{i}=\mu_{i}+x_{i}\Gamma \beta +p_{i}△\Psi b_{i}+e_{i}\;(i=1,...,n),$$

where *b_i_*=(*b*_*i*1_, ...*b_ik_*)^*T*^ such that *b_ij_*∼*N*(0, 1) and $b_{ij}⊥b_{ij}^{'}\;(j\neq j^{'})$, *j*=1, ..., *k*. For later use, we define *v_i_*=*p_i_ΔΨ*=(*v*_*i*1_, ..., *v_in_i__*)^*T*^ and *v*=diag(*v*_1_, ..., *v_n_*).

### Model identifiability

Model identifiability is a property that a model must satisfy for accurate inference to be possible. A model is identifiable if it is theoretically possible to estimate the true values of the underlying parameters of the model, while a model is non-identifiable or unidentifiable if two or more parametrizations are observationally equivalent [27]. The proposed Bayesian model has an identifiability issue associated with the covariance matrix of $y=(y_{1}^{T},\;...,\;y_{n}^{T}{)}^{T}$, which equals *PDP*^*T*^+*σ^2^I_N_* where *D*=*I_n_*⊗*D*. The condition is that $PDP^{T}+\sigma^{2}I_{N}=P{\hat{DP}}^{T}+{\hat{\sigma }}^{2}I_{N}$ if and only if $\hat{D}=D\;$ and ${\hat{\sigma }}^{2}=\sigma^{2}$. This is equivalent to the system of equations $P{\tilde{DP}}^{T}+{\hat{\sigma }}^{2}I_{N}=0$ having no non-zero solutions for $\tilde{D}$ and ${\tilde{\sigma }}^{2}$ when $\tilde{D}=D-\hat{D}$ and ${\tilde{\sigma }}^{2}=\sigma^{2}-{\hat{\sigma }}^{2}$. Let the (*r, s*)th element of $\tilde{D}$ be ${\tilde{d}}_{r,s}$. The system of equations $P{\tilde{DP}}^{T}+{\tilde{\sigma }}^{2}I_{N}=0$ is equivalent to the system of equations *AX*=0, where $A=(A_{1}^{T},\;...,\;A_{n}^{T}{)}^{T}$ is a $\left[\frac{1}{2}\sum_{i=1}^{n}n_{i}(n_{i}+1)]\times [\frac{1}{2}k(k+1)+1\right]$ matrix whose elements are functions of the *a_ijr_*s and $X=({\tilde{d}}_{1,1},\;{\tilde{d}}_{1,2},\;...,\;{\tilde{d}}_{1,\;k},\;{\tilde{d}}_{2,2},\;...,\;{\tilde{d}}_{k,k},\;{\tilde{\sigma }}^{2}{)}^{T}$ which contains all elements of the matrix $\tilde{D}$ and ${\tilde{\sigma }}^{2}$ (see proof of Lemma 1 in Supplementary Data 1). Therefore, the proposed Bayesian model (2) is identifiable if and only if rank $(A)=\frac{1}{2}k(k+1)+1$ (see proof of Theorem 1 in Supplementary Data 1).

Lemma 1 and Theorem 1 enable us to check whether a given model is identifiable. A toy example is provided below. Suppose there are 3 grid points that produce 2 time intervals. According to the theorem, the rank of *A* must be $\frac{1}{2}3(3+1)+1=7$ for the model to be identifiable. Suppose the phenotypes of all individuals are observed exactly on the 3 grid points. Then $p_{i}=I_{3},\;A_{1}=\cdots =A_{n}\;=\left(\begin{array}{ccccccc} 1 & 0 & 0 & 0 & 0 & 0 & 1\\ 0 & 1 & 0 & 0 & 0 & 0 & 0\\ 0 & 0 & 1 & 0 & 0 & 0 & 0\\ 0 & 0 & 0 & 1 & 0 & 0 & 1\\ 0 & 0 & 0 & 0 & 1 & 0 & 0\\ 0 & 0 & 0 & 0 & 0 & 1 & 1 \end{array}\right)$ and $X=\left(\begin{array}{c} {\tilde{d}}_{1,1}\\ {\tilde{d}}_{1,2}\\ {\tilde{d}}_{1,3}\\ {\tilde{d}}_{2,2}\\ {\tilde{d}}_{2,3}\\ {\tilde{d}}_{3,3}\\ {\tilde{\sigma }}^{2} \end{array}\right)$. The rank of *A* is $\frac{1}{2}3(3+1)=6$. Therefore, *PDP*^*T*^+*σ^2^I_N_* is non-identifiable. If we have one additional individual who has one phenotype measured not on any of the grid points, the model becomes identifiable since the rank of *A* now increases to 7. If we do not have any additional individuals, we can avoid the identifiability issue simply by setting *σ*^2^=0 and modeling *D* directly.

### Prior specifications

For the random effects of the proposed Bayesian model, we employ the priors presented by Chen and Dunson [26]. Specifically, independent half normal priors are imposed on the diagonal elements of *Δ* and normal priors on the lower triangular elements of *Ψ*. For the fixed effects, we straightforwardly extend the priors presented in Yi et al. [17, 18].

#### Priors on *γ* and *λ*

Let *w_a_* = *P*(*γ_a_* = 1) be the inclusion probability of the *a*th genetic effect. We assume that all inclusion probabilities are independent of each other and thus the prior of *γ* is $\prod_{a=1}^{r}\;w_{a}^{\gamma_{a}}(1-w_{a}{)}^{1-\gamma_{a}}$. The inclusion probability *w_a_* is pre-determined and can vary according to whether it corresponds to a main genetic effect, SNP-SNP interaction, or SNP-covariate interaction [17]. To specify a prior on *λ*, we assume that the locations are again independent and uniformly distributed over all SNPs. For the number of SNPs (i.e., *p*), the prior distribution of genetic variant location *λ* is therefore given by $P(\lambda )=\prod_{a=1}^{r}\;P(\lambda_{a})$.

#### Priors on b, *Δ*, and *Ψ*

In model (2), we let the distribution of each *b_ij_* independently follow a standard normal distribution. Thus, the joint prior distribution of $b=(b_{1}^{T},\;...,\;b_{n}^{T}{)}^{T}$ is $P(b)\;\underset{=}{d}\;N(0,\;I_{nk})$. As priors for *Δ* and *Ψ*, we define two vectors *δ*=(*δ_l_* ∶*l*=1, ..., *k*)^*T*^ and *ψ*=(*ψ_ml_* ∶*m*=2, ..., *k*;*l*=1, ..., *m*-1)^*T*^. The prior distribution for *δ* is $P(\delta )=\prod_{l=1}^{k}\;P(\delta_{l})\;=\;\prod_{l=1}^{k}\;N^{+}(\delta_{1}|m_{l0},\;s_{l0}^{2})$, where $N^{+}\left(\delta_{1}|m_{l0},\;s_{l0}^{2}\right)$ is the density of a half normal distribution that is a $N\left(\delta_{1}|m_{l0},\;s_{l0}^{2}\right)$ density truncated below by zero. The prior distribution for *ψ* is $P(\Psi )\;\overset{d}{=}\;N(\Psi_{0},\;R_{0})$, where *ψ*_0_ and *R*_0_ are pre-specified hyperparameters.

#### Priors on β, μ, and *σ*^2^

The prior for the *a*th genetic effect is a normal distribution, $P(\beta_{a}|\gamma_{a},\;\sigma_{\beta }^{2})\;\underset{=}{d}\;N(0,\;\gamma_{a}\sigma_{\beta }^{2})$ and the prior for the variance $\sigma_{\beta }^{2}$ is a scaled inverse *χ*^2^ distribution, $P(\sigma_{\beta }^{2})\;\underset{=}{d}\;inv-\chi^{2}(\nu_{\beta },\;s_{\beta }^{2})$ whose expectation is $E(\sigma_{\beta }^{2})\;=\frac{\nu_{\beta }s_{\beta }^{2}}{\nu_{\beta }-2}$. The degree of freedom *ν_β_* controls the skewness of the prior for $\sigma_{\beta }^{2}$ (we set *ν_β_*=6) and the scale parameter $s_{\beta }^{2}$ controls the prior confidence region for the heritability of the associated genetic factor. Let *V* be the total phenotypic variance and *V_a_* be the sample variance of the column of *x_i_* associated with *β_a_*. The heritability of the *a*th genetic factor, *h_a_*, is therefore $V_{a}\beta_{a}^{2}/V$. Setting $E(\sigma_{\beta }^{2})\;=\;E(\beta_{a}^{2})$, we have $s_{\beta }^{2}\;=\;(\nu_{\beta }-2)E(\sigma_{\beta }^{2})/\nu_{\beta }\;=(\nu_{\beta }-2)E(h_{a})V/(\nu_{\beta }V_{a})$, with E(*h_a_*)= 0.1. The prior for the overall mean μ is given by $P(\mu )\;\underset{=}{d}\;N(\eta_{0},\;\tau_{0}^{2})$. We empirically set $\eta_{0}\;=\;\bar{y}\;=\;(\frac{1}{N})\sum_{i=1}^{n}\sum_{j=1}^{n_{i}}\;y_{ij}$ and $\tau_{0}^{2}\;=\;s_{y}^{2}\;=\;(\frac{1}{N-1})\sum_{i=1}^{n}\;\sum_{j=1}^{n_{i}}\;(y_{ij}-\bar{y}{)}^{2}$. The prior for the residual variance *σ*^2^ is chosen as an scaled inverse *χ*^2^ distribution, $P(\sigma^{2})\;\underset{=}{d}\;inv-\chi^{2}(\nu_{\sigma },\;s_{\sigma }^{2})$.

### Posterior calculation and MCMC algorithm

The joint posterior distribution is proportional to the product of the likelihood and the prior distributions of all unknown parameters, which can be expressed as


(3) $$P(\gamma ,\;\theta |y)\;\propto \;P(y|\gamma ,\;\theta )P(\gamma )P(\lambda )P(\beta |\gamma )P(b)P(\delta )P(\psi )P(\mu )P(\sigma^{2}),$$

where *θ*=(*λ, β, b, δ, ψ, μ, σ*^2^)^*T*^. To obtain MCMC samples of all parameters, we utilize the Metropolis-Hastings and Gibbs sampling algorithms, and alternately update each unknown parameter or set of unknown parameters conditional on all the other parameters and the observed data.

For *γ* and *λ*, we use the Metropolis-Hastings algorithm within Gibbs sampler since their conditional distributions have no known distributional forms. To update those parameters, we straightforwardly extend the Metropolis-Hastings algorithm proposed by Yi et al. [18] for our Bayesian model. These algorithms are described in the Supplementary Data 1. For the other parameters, we applied the Gibbs sampling algorithm. Specifically, since *b*, *δ*, and *ψ* have multivariate normal or half normal priors, the full conditional distributions are easy to derive by their conjugacy properties. The full conditional posterior distributions of *b*, *δ* and *ψ* are $P(b|y,\;\gamma ,\;\theta_{-b})\;\underset{=}{d}\;N(b^{*},\;\sum_{b}^{*})$, $P(\delta_{l}|y,\;\gamma ,\;\theta_{-\delta_{l}})\;\underset{=}{d}\;N^{+}(\delta_{l}^{*},\;\sigma_{l}^{{*}^{2}})$, and $P(\psi |y,\gamma ,\theta_{-\psi })\;\underset{=}{d}\;N(\psi^{*},\;\sum_{\psi }^{*})$, respectively, where *θ*_-*f*_ represents all the elements of *θ* except *f*. The expressions for b^*^, $\sum_{b}^{*}$, $\delta_{l}^{*}$, $\delta_{l}^{{*}^{2}}$, ψ^*^, and $\sum_{\psi }^{*}$ are again given in Supplementary Data 1. The full conditional distributions of *β*, $\sigma_{\beta }^{2}$, *μ* and *σ*^2^ are $P(\beta_{a}|\gamma_{a}=1,\;\gamma_{-a},\;\theta_{-\beta_{a}},\;y)\;\underset{=}{d}\;N({\tilde{\mu }}_{a},\;{\tilde{\sigma }}_{\beta }^{2})$, $P(\sigma_{\beta }^{2}|\beta_{a})\;\underset{=}{d}\;Inv-\chi^{2}(\nu_{\beta }+1,\;(\beta_{a}^{2}+\nu_{\beta }s_{\beta }^{2})/(\nu_{\beta }+1))$, $P(\mu |\gamma ,\;\theta_{-\mu },\;y)\;\underset{=}{d}\;(\mu^{*},\;\sigma_{\mu }^{2*})$, and $P(\sigma^{2}|\gamma ,\;\theta_{-\sigma^{2}},\;y)\;\underset{=}{d}\;Inv-\chi^{2}(\nu_{\sigma }+N,\;\frac{\nu_{\sigma }s_{\sigma }^{2}+N{\hat{\sigma }}^{2}}{\nu_{\sigma }+N})$, respectively, where ${\tilde{\mu }}_{a}$, ${\tilde{\sigma }}_{\beta }^{2}$, μ^*^, $\sigma_{\mu }^{2*}$, and ${\hat{\sigma }}^{2}$ are given in Supplementary Data 1 as well.

### Posterior analysis

The posterior samples can be used to approximate the posterior distribution of the parameters. MCMC samples from the initial iterations are discarded as “burn-in” and the subsequent samples are thinned by keeping every *c*th MCMC sample, where *c* is an integer, and discarding the rest. The posterior inclusion probability of each SNP can be calculated using its inclusion proportion in the MCMC samples as $P(\kappa_{l}|y)\;=\;\frac{1}{T}\sum_{t=1}^{T}\;\sum_{w=1}^{r}\;1(\lambda_{w}^{\left(t\right)}\;=\;\kappa_{l},\;\gamma_{w}^{\left(t\right)}\;=\;1)$ where *κ_l_* is *l*th SNP position (*l*=1, ..., *h*) and *T* is the total number of MCMC samples. With the prior $P(\kappa_{l})\;=\;\frac{p}{h}$, the Bayes factor can be calculated to quantify the evidence for inclusion of the *l*th SNP (*κ_l_*) against exclusion of the *l*th SNP as


(4) $$BF(\kappa_{l})\;=\;\frac{P(\kappa_{l}|y)/P(\kappa_{l})}{\left(1-P(\kappa_{l}|y))/(1-P(\kappa_{l})\right)}\;=\;\frac{P(\kappa_{l}|y)}{1-P(\kappa_{l}|y)}\frac{1-P(\kappa_{l})}{P(\kappa_{l})}$$

The Bayes factor *BF*(*κ_l_*) reflects how our belief in the importance of the *l*th SNP changes as we move from prior knowledge to posterior information. Jeffreys [28] and Yandell et al. [29] suggest the following criteria for judging the significance of each SNP: weak support if *BF*(*κ_l_*) falls between 3 and 10; moderate support if *BF*(*κ_l_*)falls between 10 and 30; and strong support if *BF*(*κ_l_*) is larger than 30.

### Choice of the number of grid points

A critical issue with the proposed Bayesian model is how to choose an optimal number of grid points, *k*. We achieve this goal by evaluating the goodness of the predictive distributions of our Bayesian models. Spiegelhalter et al. [30] proposed the DIC as $DIC\;=\;-2E_{\gamma ,\theta |y}[logP(y|\gamma ,\;\theta )]\;+\;P_{D}$. The second term of the DIC, *P_D_*, is the effective number of parameters, which is defined as $P_{D}\;=\;-2E_{\gamma ,\theta |y}\;[log\;P(y|\gamma ,\theta )]\;+\;2log\;P(y|\bar{\gamma },\;\bar{\theta })$, where $\bar{\gamma }$ and $\bar{\theta }$ are the posterior means of *γ* and *θ*. Since $P(y_{i}|\gamma ,\;\theta )\;\underset{=}{d}\;N(\mu_{i}+x_{i}\Gamma \beta ,\;p_{i}Dp_{i}^{T}\;+\;\sigma^{2}\;{I_{n}}_{i})$ in model (1), the DIC is easy to compute with the MCMC samples. However, as stated by Robert and Titterington [31], the observed data are used twice to calculate *P_D_*, and thus the predictive distribution from the DIC tends to overfit the data. To overcome the overfitting problem, Ando [32] developed the BPIC, which is defined as $BPIC\;=\;-2E_{\gamma ,\theta |y}[logP(y|\gamma ,\theta )]+2n\hat{b}$ where $\hat{b}$ is the asymptotic bias in the posterior mean of the expected log-likelihood. Under a certain mild regularity condition, the bias term can be approximated by $n\hat{b}\approx P_{D}$, resulting in the simplified $BPIC\;=\;2E_{\gamma ,\theta |y}[logP(y|\gamma ,\;\theta )]+2P_{D}$. It should be noted that the penalty term of the simplified BPIC is twice that of the original DIC. We select the optimal number of grid points for our model by minimizing DIC or simplified BPIC scores.

### Implementation in gridbayes

The proposed grid-based Bayesian mixed models have been implemented in an R package named gridbayes [33], which is built on top of the R packages, qtl [34] and qtlbim [29]. The MCMC algorithm in C and the data manipulation procedure in R were modified for longitudinal analysis. The gridbayes package employs both DIC and simplified BPIC scores to select the optimal number of grid points. The software package and the source code are available for download at https://github.com/wonilchung/GridBayes.

## Results

### Simulation I

To evaluate the performance of the proposed method, we conducted the following simulations. We first used 400 individuals and 1, 000 SNPs from the 1000 Genome Project data. The number of measurements for each individual ranged from 3 to 7 and the total number of observations was set to 2, 000. Six different setups (Setups 1‒6) were considered. We simulated the datasets containing 10 causal SNPs, which are randomly selected (i.e., the proportion of causal SNPs = 1%) with only main effects (Setup 1). For individual *i*, the phenotype values were generated from the model: $y_{i}\;=\;c_{g1}\;\cdot \;(\sum_{a=1}^{10}\;x_{ia}\;+\;t_{i})\;+\;p_{i}v_{i}\;+\;e_{i}$, where *x_ia_* (a=1, ..., 10) were genotype values of the causal SNPs, *c*_*g*1_ is used to set trait-heritability to 40%, *t_i_*=(*t*_*i*1_, ..., *t_in_i__*)^*T*^ were the time covariates generated from the uniform distribution *U*[0, 1] and then standardized to have mean 0 and variance 1, and *e_i_*∼*N*(0, *σ*^2^*I_n_i__*). We set *σ*^2^ =1. The true number of grid points was set to 3 (i.e., true *k*=3), and *p_i_* was calculated from *t_i_* by the linear interpolation as we described in the Methods section. We set *δ*=(*δ*_1_, *δ*_2_, *δ*_3_) = (1, 1.2, 0.8) and *ψ*=(*ψ*_21_, *ψ*_31_, *ψ*_32_)=(0.6, 0.4, 0.6). That is, *ν_i_*∼*N*(0, *D*) with *diag*(*D*)=(1, 1.96, 0.97) and the lower triangle elements (*d*_21_, *d*_31_, *d*_32_)=(0.72, 0.32, 0.81). The prior distributions for the elements in *δ* were independent *N*^+^ (0, 30) and the prior distributions for the elements in *ψ* were independent *N*(0, 0.5). For each simulated dataset, the MCMC algorithm ran for 4 × 10^5^ iterations after discarding the first 1, 000 burn-in iterations. The remaining samples were further thinned for every 40 iterations, yielding 10^4^ MCMC samples for the posterior analysis.

To further investigate the Bayesian mixed model, we analyzed additional datasets containing two SNP-SNP interactions (Setup 2), five SNP-SNP interactions (Setup 3), two SNP-time interactions (Setup 4), five SNP-time interactions (Setup 5), or ten SNP-time interactions (Setup 6). Specifically, we simulated data according to the following models: $y_{i}\;=\;c_{g2}\;\cdot \;(\sum_{a=1}^{6}\;x_{ia}\;+x_{i7}\;\cdot \;x_{i8}\;+\;x_{i9}\cdot x_{i10}\;+\;t_{i})\;+\;p_{i}v_{i}\;+\;e_{i}$ for Setup 2, $y_{i}\;=\;c_{g3}\;\cdot (x_{i1}\cdot x_{i2}\;+\;x_{i3}\cdot x_{i4}\;+\;x_{i5}\cdot x_{i6}+\;x_{i7}\cdot x_{i8}\;+\;x_{i9}\cdot x_{i10}\;+\;t_{i})\;+\;p_{i}v_{i}\;+\;e_{i}$ for Setup 3, $y_{i}\;=\;c_{g4}\;\cdot \;(\sum_{a=1}^{8}\;x_{ia}\;+\;\sum_{a=9}^{10}\;x_{ia}\cdot t_{i})\;+\;p_{i}v_{i}\;+\;e_{i}$ for Setup 4, $y_{i}\;=\;c_{g5}\;\cdot (\sum_{a=1}^{5}\;x_{ia}\;+\;\sum_{a=6}^{10}\;x_{ia}\cdot t_{i})\;+\;p_{i}v_{i}\;+\;e_{i}$ for Setup 5 and $y_{i}\;=\;c_{g6}\;\cdot \;(\sum_{a=1}^{10}\;x_{ia}\cdot t_{i})\;+\;p_{i}v_{i}\;+\;e_{i}$ for Setup 6. In our simulations, *c_gj_* (j = 1, ..., 6) were varied to ensure that trait-heritability to 40%. To display time-dependent SNP effects for Setups 4 and 5, we compared the time-dependent curves of averaged phenotype values for three different genotypes (0, 1, 2) at the first causal SNP (with no SNP-time interaction) and 10th one (with SNP-time interaction). Supplementary Fig. 1 clearly showed that the first causal SNP had only a main effect, but the 10th causal SNP interacted with time. We first conducted gridbayes [33] using all the data. For model comparisons, we then conducted qtlbim [29] in two ways: once on a subset of each simulated data, where only one measurement from each subject was randomly selected, and once with all the data by (incorrectly) assuming that all the measurements were independent. We named the two qtlbim analyses “qtlbim-sub” and “qtlbim-all, ” respectively.

**Fig. 1.**
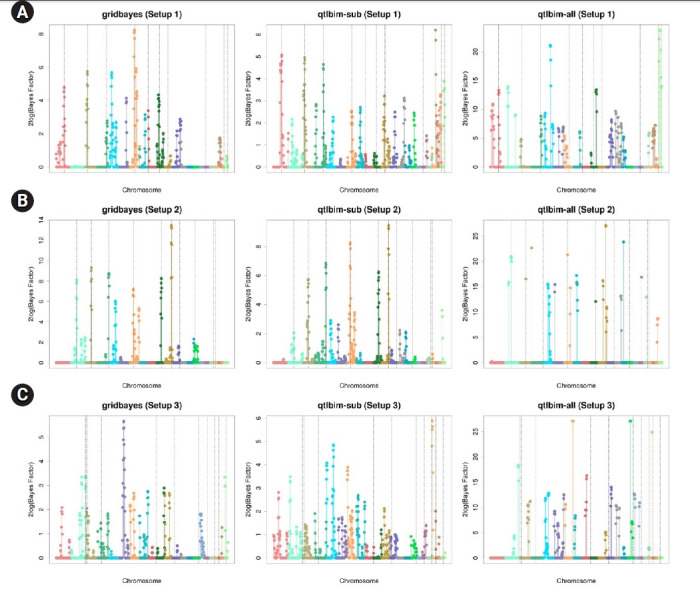
Genome-wide profiles of 2*log*(*BF*) for all combined effects using gridbayes with all time points, qtlbim with one randomly-selected time point (qtlbim-sub) and qtlbim with all time points (qtlbim-all) for Setups 1 (A), 2 (B), and 3 (C).

**Fig. 2.**
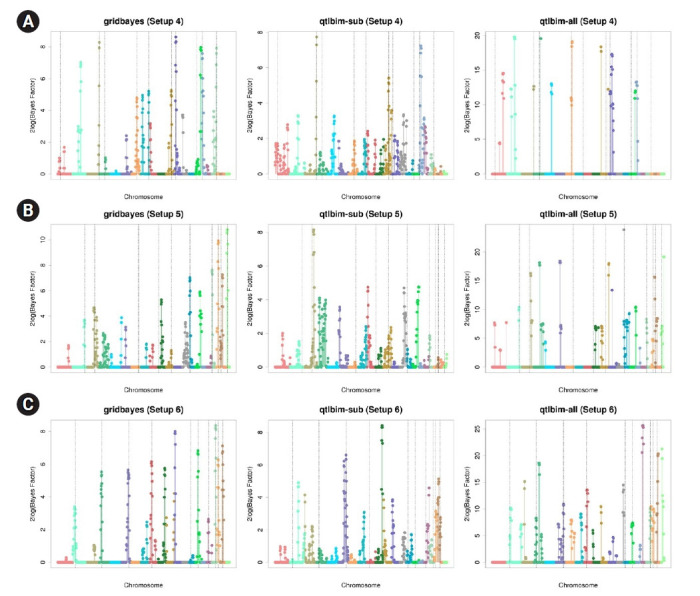
Genome-wide profiles of 2*log*(*BF*) for all combined effects using gridbayes with all time points, qtlbim with one randomly-selected time point (qtlbim-sub) and qtlbim with all time points (qtlbim-all) for Setups 4 (A), 5 (B), and 6 (C).

The one-dimensional genome-wide profiles of 2*log*(*BF*) for the combined main, epistatic effects, and SNP-time interactions of each SNP under the six setups were presented in Figs. 1 and 2. The dashed vertical lines indicate the locations of the 10 causal SNPs. The gridbayes analysis of all the data and qtlbim-sub detected the causal SNPs reasonably well, but gridbayes clearly outperformed qtlbim-sub in general. The qtlbim-all method occasionally identified the true causal SNPs, but it produced far more false-positive findings than gridbayes and qtlbim-sub.

**Fig. 3.**
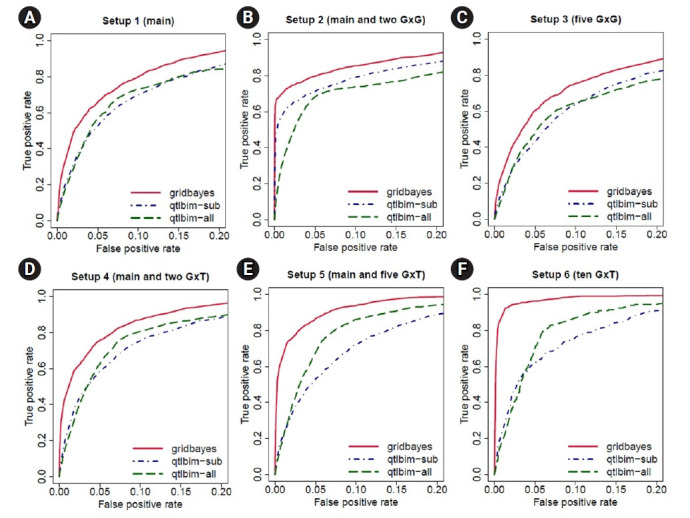
Receiving operating characteristic curve analyses in the simulation study for Setup 1 (A), 2 (B), 3 (C), 4 (D), 5 (E) and 6 (F). The red solid lines represent the results of gridbayes, the blue dot-dashed lines correspond to qtlbim-sub and the green long-dashed lines display the results from qtlbim-all. gridbayes: grid-based Bayesian mixed models with all the data; qtlbim-sub qtlbim with a subset of each simulated data where only one measurement from each subject was randomly selected; qtlbim-all: qtlbim with all the data by (incorrectly) assuming that all the measurements were independent. G, gene; T, time.

To evaluate the performance of our Bayesian model, we further calculated the receiver operating characteristic (ROC) curves. For each setup, we conducted 100 simulations. The ROC curves with a false-positive rate less than 0.2 are presented in Fig. 3. The solid lines represent the results of gridbayes, the dot-dashed lines correspond to qtlbim-sub and the results from qtlbim-all are summarized by the long-dashed lines. The ROC curves demonstrated that gridbayes with all measurements appeared to outperform the qtlbim analyses in terms of improved true positive rates.

To diagnose the convergence of the MCMC samples, we conducted 10 parallel chains with different, over-dispersed initial values with respect to the true posterior distribution. Using 10^4^ iterations, Geweke's Z-scores [35] for each chain based on the first 10% and last 50% of the samples indicated good convergence of all parameters. Based on 10 chains, Gelman and Rubin's potential scale reduction factors [36] were calculated, and the upper limits were less than 1.01 for all parameters. Supplementary Fig. 2 presents the trace plots of *σ*^2^, *δ*_1_, *δ*_2_, *δ*_3_, *ψ*_21_, *ψ*_31_ and *ψ*_32_ for each setup, showing that all chains moved around the true values for all parameters, indicating good convergence. We plotted the marginal posterior and prior densities of all parameters based on 10, 000 random draws (Supplementary Fig. 3). It appeared that the random draws were approximately normal, with means close to the simulated values. Supplementary Fig. 4 displays the 95% highest posterior density (HPD) intervals for *σ*^2^, *δ*_1_, *δ*_2_, *δ*_3_, *ψ*_21_, *ψ*_31_ and *ψ*_32_ for each setup. Most of the 95% HPD intervals contained the corresponding true values. Table 1 summarizes the posterior estimates of all parameters. The posterior means and medians were close to the true values and all the 95% HPD intervals contained the true values, demonstrating the good performance of our algorithm.

### Simulation II

We conducted another simulation to estimate the number of true grid points using the DIC [30] and simplified BPIC [32,37]. The settings were almost the same as those in the previous simulations, except that the true number of grid points now varied from 2 to 4 (i.e., true *k*=2, 3, 4). We simulated 100 datasets with 400 individuals and 1, 000 SNPs containing 10 causal SNPs (i.e., the proportion of causal SNPs = 1%) with only main effects. The causal SNPs were randomly assigned. The trait-heritability was set to 40%. The phenotype values were generated from the model: $y_{i}\;=\;c_{g1}\;\cdot \;(\sum_{a=1}^{10}\;x_{ia}\cdot t_{i})\;+\;p_{i}v_{i}\;+\;e_{i}$, where *x_ia_* (*a*=1, ..., 10) are genotypes of the causal SNPs and *t_i_*=(*t*_*i*1_, ..., *t_in_i__*)^*T*^ are the time points of the *i*th individual. We set (*δ*_1_, *δ*_2_, *δ*_3_, *δ*_4_)=(1, 1.2, 0.8, 0.7) and (*ψ*_21_, *ψ*_31_, *ψ*_32_, *ψ*_41_, *ψ*_42_, *ψ*_43_)=(0.6, 0.4, 0.6, 0.2, 0.4, 0.6). Table 2 shows the average DIC, simplified BPIC scores over 100 simulations, and the proportion of times that the number of true grid points was correctly selected. All average DIC and average BPIC scores achieved the minimums at the true grid point number, and the percentages correctly selecting the true number of true grid points were 79%, 91%, and 100% for setups with 2, 3, and 4 true grid points using the DIC, and 94%, 98%, and 93% using the simplified BPIC. This illustrated the usefulness of the DIC and simplified BPIC in selecting the true number of grid points.

### Simulation III

**Fig. 4.**
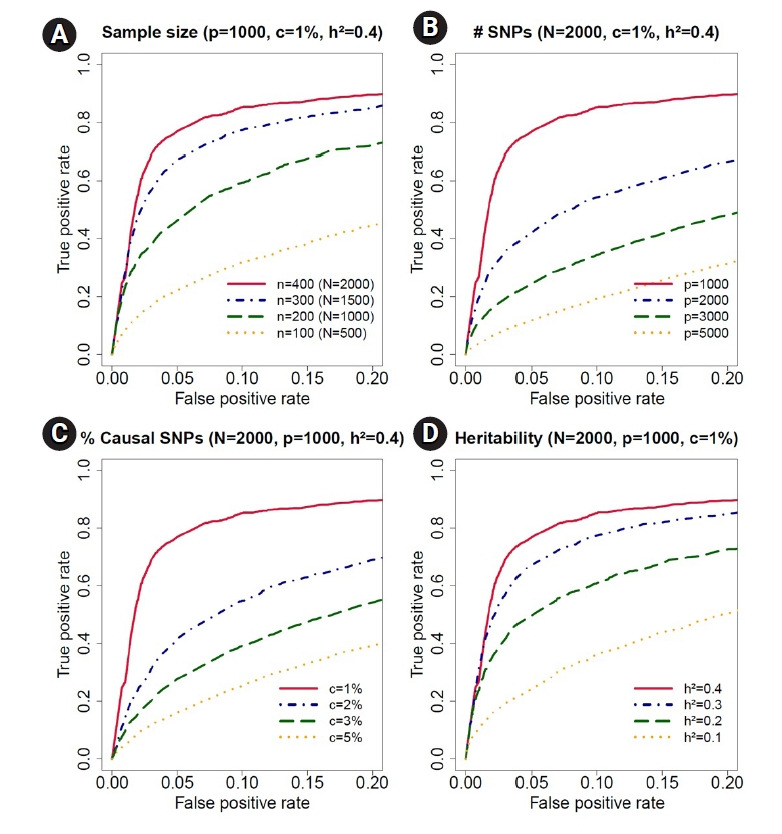
Receiving operating characteristic curve analyses in the simulation study for sample size, number of single-nucleotide polymorphisms (SNPs), proportion of causal SNPs, and heritability. (A) We decreased the sample size from *n* = 400 (total number of observations, N = 2, 000) to *n* = 100 (N = 1, 000) for accessing the effect of sample size in receiver operating characteristic curves. The simulation data contained *p* = 1, 000 SNPs, c = 1% causal SNPs and h^2^ = 40% trait-heritability. (B) We increased the number of SNPs from *p* = 1, 000 to *p* = 5, 000 to evaluate the effect of number of SNPs. The simulation data contained N = 2, 000 observations, c = 1% causal SNPs, and h^2^ = 40% trait-heritability. (C) We increased the proportion of causal SNPs from c = 1% to 5%. The simulation data contained N = 2, 000 observations, *p* = 1, 000 SNPs, and h^2^ = 40% trait-heritability. (D) We decreased the trait-heritability from h^2^ = 40% to h^2^ = 10%. The simulation data contained N = 2, 000 observations, *p* = 1, 000 SNPs, and c = 1% causal SNPs.

For a more detailed evaluation of our Bayesian method, we conducted the following simulations with 100 replications for each scenario. We first considered 400 individuals with three to seven time points, resulting in 2,000 observations, and decreased the sample size from 400 to 100 to assess the effect of sample size in ROC curves (Fig. 4A). The simulation data contained 1,000 SNPs with 1% causal SNPs (i.e., 10 causal SNPs) with only main effects. The trait values were generated from the model: $y_{i}\;=\;c_{g1}\;\cdot \;(\sum_{a=1}^{10}\;x_{ia}\cdot t_{i})\;+\;p_{i}v_{i}\;+\;e_{i}$, where *x_ia_* are genotypes of the causal SNPs and *t_i_*=(*t*_*i*1_, ..., t_(i*n_i_*))^*T*^ are the time points of the *i*th individual. As in the previous simulations, we set the number of grid points to *k* = 3 and *σ*^2^ = 1, *δ*=(*δ*_1_, *δ*_2_, *δ*_3_) = (1, 1.2, 0.8), *ψ*=(*ψ*_21_, *ψ*_31_, *ψ*_32_)=(0.6, 0.4, 0.6). The trait-heritability was set to 40%. As the sample size decreased from 400 to 100, the true positive rates decreased in ROC curves, indicating that including more samples increased the true positive rates with fixed false positive rates. Next, we evaluated the effect of the number of SNPs (Fig. 4B). The simulation data were generated with 400 individuals, 1% causal SNPs, and 40% trait-heritability. As the number of SNPs increased from 1,000 to 5,000 (i.e., the corresponding number of causal SNPs increased from 10 to 50), the true positive rates decreased, meaning that the inclusion of more SNPs deceased the true positive rates. We then examined the effect of the proportion of causal SNPs (Fig. 4C). The sample size and number of SNPs were fixed to 400 and 1,000, and the trait-heritability was set to 40%. The true positive rates decreased as the proportion of causal SNPs increased from 1% to 5% (i.e., the corresponding number of causal SNPs increased from 10 to 50) because per-SNP heritability—or the average proportion of phenotypic variation explained by a single SNP—decreased as the proportion of causal SNPs increased while keeping trait-heritability constant. Lastly, to demonstrate the effect of trait-heritability, we considered a setting where the sample size, number of SNPs, and proportion of causal SNPs were 400, 1,000, and 1%, respectively. We then changed trait-heritability from 40% to 10% in Fig. 4D. The true-positive rates decreased as trait-heritability decreased, showing that larger heritability increased the true positive rates. Supplementary Tables 1, 2, 3, and 4 summarize the posterior means, medians, standard deviations and 95% HPD intervals of all parameters in the simulations for sample size, number of SNPs, proportion of causal SNPs, and heritability, respectively. The posterior means and medians were close to the true values, and all the 95% HPD intervals contained the true values, indicating that our Bayesian method performed well. Supplementary Table 5 showed the average DIC and simplified BPIC scores over 100 replications for all simulations. Table 3 summarizes the simulation settings for all simulation setups based on genetic effect terms, the number of grid points, sample size, number of observations, number of SNPs, number of causal SNPs, and trait-heritability.

## Discussion

We developed a grid-based Bayesian mixed model for longitudinal genetic data with a built-in variable selection feature. The proposed Bayesian method modeled multiple candidate SNPs simultaneously and allowed SNP-SNP and SNP-time interactions, which enabled us to identify SNPs with time-varying effects. Such SNPs are of great scientific and medical interest. In addition, we proposed a new grid-based method to model the covariance structure nonparametrically. Not only is the proposed method parsimonious in estimating the covariance matrix, but also by employing a reasonable number of grid-points, it can flexibly approximate any type of covariance structure. The number of grid points was pre-set, but DIC and simplified BPIC can be used to select the optimal number.

The simulation studies showed that the proposed Bayesian method using all time points outperformed the ordinary Bayesian method with one or all time points included. As expected, the proposed method that utilized the full data was more powerful than the corresponding univariate analysis method that only used a subset of the data. Furthermore, the proposed Bayesian method performed better than the ordinary Bayesian method because our method modeled the within-subject correlation. Further simulation studies showed that statistical power increased as the data had more samples, a smaller number of SNPs, a lower proportion of causal SNPs, and larger trait-heritability. For our simulation studies, we utilized data from the 1000 Genome Project. With only 400 independent samples of EAS ancestry, we restricted out analysis with up to 5, 000 SNPs. With a sufficient sample size, our method can be applied to all available SNPs. We are currently developing a parallel computing algorithm based on the message passing interface to execute multiple groups of SNPs simultaneously. This will make it feasible to apply our method to large-sample GWAS data.

Another important issue to mention is Bayesian model identifiability. In the Bayesian community, there is a wide diversity of views on the identifiability issue. Lindley [38] remarked that non-identifiability causes no real difficulty in Bayesian approaches. Poirier [39] and Eberly and Carlin [40] argued that a Bayesian analysis of a non-identifiable model is always possible if priors on all of the parameters are proper, since proper priors yield proper posterior distributions, and hence every parameter can be well-estimated. However, if the priors imposed on any non-identifiable model are not proper, or too close to being improper, ill-behaved posterior distributions may be generated such that the trajectory of the parameters can drift to extreme values, as demonstrated by Gelfand and Sahu [41]. In this paper, we investigated the identifiability of our Bayesian model, which motivated us to utilize only proper priors (see the Methods section). Non-identifiability occurred when the number of the grid points equaled the number of observed time points (see Supplementary Data 1), but we found that the posterior distribution behaved well due to the proper priors employed.

## Figures and Tables

**Table 1.** Posterior means, medians, standard deviations, and 95% HPD intervals of the parameters for random errors and random effects in the simulation study

Setup	Par	True	Mean	Med	SD	95% HPD	Setup	Par	True	Mean	Med	SD	95% HPD
1	*σ* ^2^	1	1.01	1.01	0.04	0.92 to 1.09	2	*σ* ^2^	1	1.01	1.01	0.04	0.93 to 1.10
	*δ*_1_	1	0.98	0.98	0.11	0.77 to 1.19		*δ*_1_	1	0.98	0.98	0.10	0.78 to 1.19
	*δ*_2_	1.2	1.19	1.19	0.13	0.92 to 1.43		*δ*_2_	1.2	1.19	1.19	0.13	0.92 to 1.43
	*δ*_3_	0.8	0.76	0.76	0.13	0.5 to 1.00		*δ*_3_	0.8	0.72	0.73	0.13	0.48 to 0.97
	*ψ*_21_	0.6	0.65	0.63	0.21	0.31 to 1.13		*ψ*_21_	0.6	0.67	0.64	0.21	0.33 to 1.14
	*ψ*_31_	0.4	0.52	0.51	0.24	0.09 to 1.04		*ψ*_31_	0.4	0.51	0.49	0.24	0.07 to 1.01
	*ψ*_32_	0.6	0.56	0.53	0.27	0.11 to 1.18		*ψ*_32_	0.6	0.67	0.64	0.29	0.20 to 1.34
3	*σ* ^2^	1	1.00	1.00	0.04	0.92 to 1.08	4	*σ* ^2^	1	1.00	1.00	0.04	0.92 to 1.09
	*δ*_1_	1	1.06	1.06	0.10	0.85 to 1.26		*δ*_1_	1	0.99	0.99	0.10	0.78 to 1.19
	*δ*_2_	1.2	1.20	1.20	0.13	0.94 to 1.44		*δ*_2_	1.2	1.18	1.19	0.13	0.92 to 1.42
	*δ*_3_	0.8	0.74	0.74	0.12	0.49 to 0.98		*δ*_3_	0.8	0.74	0.74	0.13	0.48 to 0.98
	*ψ*_21_	0.6	0.69	0.67	0.20	0.36 to 1.14		*ψ*_21_	0.6	0.62	0.60	0.20	0.29 to 1.07
	*ψ*_31_	0.4	0.65	0.64	0.24	0.22 to 1.17		*ψ*_31_	0.4	0.47	0.45	0.24	0.03 to 0.96
	*ψ*_32_	0.6	0.65	0.62	0.27	0.19 to 1.27		*ψ*_32_	0.6	0.65	0.61	0.29	0.18 to 1.31
5	*σ* ^2^	1	1.00	1.00	0.04	0.92 to 1.09	6	*σ* ^2^	1	1.00	1.00	0.04	0.92 to 1.09
	*δ*_1_	1	0.98	0.98	0.10	0.77 to 1.18		*δ*_1_	1	0.96	0.96	0.11	0.75 to 1.17
	*δ*_2_	1.2	1.20	1.20	0.13	0.93 to 1.43		*δ*_2_	1.2	1.18	1.19	0.13	0.92 to 1.41
	*δ*_3_	0.8	0.72	0.72	0.13	0.47 to 0.97		*δ*_3_	0.8	0.75	0.75	0.13	0.48 to 1.01
	*ψ*_21_	0.6	0.63	0.60	0.20	0.29 to 1.09		*ψ*_21_	0.6	0.61	0.58	0.20	0.27 to 1.07
	*ψ*_31_	0.4	0.46	0.45	0.25	0.01 to 0.99		*ψ*_31_	0.4	0.32	0.31	0.24	–0.14 to 0.81
	*ψ*_32_	0.6	0.65	0.61	0.29	0.18 to 1.31		*ψ*_32_	0.6	0.67	0.63	0.30	0.19 to 1.35

HPD, highest posterior density; Par, parameters; True, true values of parameters; Med, median; SD, standard deviation.

**Table 2.** Average DIC scores and simplified BPIC scores over 100 replications and the proportion selecting the model with the correct number of grid points using the proposed Bayesian model

True k	k	Avg DIC	#Sel (%)	Avg Sim BPIC	#Sel (%)	Avg *P_D_*
2	2	6,614.47	79	6,681.78	94	67.32
	3	6,617.97	15	6,690.51	6	72.54
	4	6,623.02	6	6,700.99	0	77.98
3	2	6,745.26	0	6,812.49	0	67.23
	3	6,696.53	91	6,769.83	98	73.30
	4	6,707.12	9	6,785.14	2	78.02
4	2	6,745.07	0	6,814.76	0	69.70
	3	6,718.66	0	6,792.75	7	74.09
	4	6,695.41	100	6,775.37	93	79.96

DIC, deviance information criterion; BPIC, Bayesian predictive information criterion; Avg DIC, average deviance information criterion scores over 100 replications; #Sel (%), proportion selecting the model with the correct number of grid points; Avg Sim BPIC, average simplified Bayesian predictive information criterion scores over 100 replications; Avg PD, average *P_D_*.

**Table 3.** Simulation settings for all simulation setups in the Results section based on genetic effect terms, the number of grid points (k), sample size (n), number of observations (N), number of SNPs (p), number of causal SNPs (c), and trait-heritability (h^2^)

Simulation	Setup	Genetic effect terms	k	n	N	p	c (%)	h^2 ^(%)
I	1	Only main effects	3	400	2,000	,000	1	40
	2	Two SNP-SNP interactions	3	400	2,000	1,000	1	40
	3	Five SNP-SNP interactions	3	400	2,000	1,000	1	40
	4	Two SNP-time interactions	3	400	2,000	1,000	1	40
	5	Five SNP-time interactions	3	400	2,000	1,000	1	40
	6	Ten SNP-time interactions	3	400	2,000	1,000	1	40
II	1	Only main effects	2	400	2,000	1,000	1	40
	2	Only main effects	3	400	2,000	1,000	1	40
	3	Only main effects	4	400	2,000	1,000	1	40
III (a)	1	Only main effects	3	300	1,500	1,000	1	40
	2	Only main effects	3	200	1,000	1,000	1	40
	3	Only main effects	3	100	500	1,000	1	40
III (b)	1	Only main effects	3	400	2,000	2,000	1	40
	2	Only main effects	3	400	2,000	3,000	1	40
	3	Only main effects	3	400	2,000	5,000	1	40
III (c)	1	Only main effects	3	400	2,000	1,000	2	40
	2	Only main effects	3	400	2,000	1,000	3	40
	3	Only main effects	3	400	2,000	1,000	5	40
III (d)	1	Only main effects	3	400	2,000	1,000	1	40
	2	Only main effects	3	400	2,000	1,000	1	30
	3	Only main effects	3	400	2,000	1,000	1	20

SNP, single nucleotide polymorphism.

## Supplementary Materials

Supplementary Data 1

Supplementary Fig. 1. Time-dependent curves of averaged phenotype values for three different genotypes (0, 1, 2) at 1st causal single nucleotide polymorphism (SNP) with no SNP-time interaction and 10th causal SNP with SNP-time interaction for Setups 4 and 5.

Supplementary Fig. 2. Trace plots of *σ*^2^, *δ*_1_, *δ*_2_, *δ*_3_, *ψ*_21_, *ψ*_31_ and *ψ*_22_ for Setups 1‒6 in the simulation study.The black lines represent the values of the draws for all parameters at each iteration and gray lines represent the true values of the parameters.

Supplementary Fig. 3. Posterior (solid line) and prior (dashed line) densities of the parameters for random errors and random effects for Setups 1‒6. Estimated densities are based on 10, 000 random draws.

Supplementary Fig. 4. 95% highest posterior density (HPD) intervals for *σ*^2^, *δ*_1_, *δ*_2_, *δ*_3_, *ψ*_21_, *ψ*_31_ and *ψ*_22_ for Setups 1‒6. The blue lines represent the 95% HPD intervals (100 replicates).

Supplementary Table 1. Posterior means, medians, standard deviations and 95% HPD intervals of the parameters for random errors and random effects in the simulation study for sample size

Supplementary Table 2. Posterior means, medians, standard deviations, and 95% HPD intervals of the parameters for random errors and random effects in the simulation study for number of SNPs

Supplementary Table 3. Posterior means, medians, standard deviations, and 95% HPD intervals of the parameters for random errors and random effects in the simulation study for proportion of causal SNPs

Supplementary Table 4. Posterior means, medians, standard deviations, and 95% HPD intervals of the parameters for random errors and random effects in the simulation study for heritability

Supplementary Table 5. Average DIC scores and simplified BPIC scores over 100 replications in the simulation study for sample size, number of SNPs, proportion of causal SNPs and heritability
