# Supplementary material for: Bayesian mixed models for longitudinal genetic data: theory, concepts, and simulation studies
Source: Genomics Inform. 2022 Mar 31;20(1):e8. doi: 10.5808/gi.21080 (PMC9001998; doi:10.5808/gi.21080)
Supplement: Supplementary Data 1 [file gi-21080suppl1.pdf]

Supplementary Data 1

Theorem on Model Identifiability

Lemma 1 The system of equations $\boldsymbol{P}\widetilde{\boldsymbol{\mathcal{D}}}\boldsymbol{P}^T + \tilde{\sigma}^2\boldsymbol{I}_N = \boldsymbol{0}$ where $\widetilde{\boldsymbol{\mathcal{D}}} = \boldsymbol{\mathcal{D}} - \widehat{\boldsymbol{\mathcal{D}}}$ and $\tilde{\sigma}^2 = \sigma^2 - \hat{\sigma}^2$ is equivalent to the system of equations $\boldsymbol{AX} = \boldsymbol{0}$ where $\boldsymbol{A} = (\boldsymbol{A}_1^T, \ldots, \boldsymbol{A}_n^T)^T$ is a $\left[\frac{1}{2}\sum_{i=1}^{n} n_i(n_i+1)\right] \times [\frac{1}{2}k(k+1)+1]$ matrix whose elements are functions of a_{ijr}s (see proof below for details), and $\boldsymbol{X} = \left(\tilde{d}_{1,1}, \tilde{d}_{1,2}, \cdots, \tilde{d}_{1,k}, \tilde{d}_{2,2}, \cdots, \tilde{d}_{k,k}, \tilde{\sigma}^2\right)^T$ which contains all elements of matrix $\widetilde{\boldsymbol{\mathcal{D}}}$ and $\tilde{\sigma}^2$.

(Proof) Since only one or two adjacent a_{ijr} are nonzero, $\boldsymbol{p}_{ij}$ can be expressed as $\boldsymbol{p}_{ij} = a_{ij(c_{ij})}\boldsymbol{e}_{(c_{ij})} + a_{ij(c_{ij}+1)}\boldsymbol{e}_{(c_{ij}+1)}$ where $[c_{ij,}\ c_{ij}+1]$ refers the time interval on which the jth time of the ith subject falls and $1 \le c_{ij} \le k-1$. Note the (j,j') th element of $\boldsymbol{p}_i\widetilde{\boldsymbol{D}}\boldsymbol{p}_i^T + \tilde{\sigma}^2\boldsymbol{I}_{n_i}$ equals $\boldsymbol{p}_{ij}\widetilde{\boldsymbol{D}}\boldsymbol{p}_{ij'}^T + \tilde{\sigma}^2 I(j=j')$, and $\boldsymbol{e}_r\widetilde{\boldsymbol{D}}\boldsymbol{e}_s^T = \tilde{d}_{r,s}$ $(1 \le r,s \le k)$. Thus $\boldsymbol{p}_{ij}\widetilde{\boldsymbol{D}}\boldsymbol{p}_{ij'}^T + \tilde{\sigma}^2 I(j=j') = a_{ij(c_{ij})}a_{ij'(c_{ij'})}\boldsymbol{e}_{(c_{ij})}\widetilde{\boldsymbol{D}}\boldsymbol{e}_{(c_{ij'})}^T + a_{ij(c_{ij})}a_{ij'(c_{ij'}+1)}\boldsymbol{e}_{(c_{ij})}\widetilde{\boldsymbol{D}}\boldsymbol{e}_{(c_{ij'}+1)}^T + a_{ij(c_{ij}+1)}a_{ij'(c_{ij'})}\boldsymbol{e}_{(c_{ij}+1)}\widetilde{\boldsymbol{D}}\boldsymbol{e}_{(c_{ij'})}^T + a_{ij(c_{ij}+1)}a_{ij'(c_{ij'}+1)}\boldsymbol{e}_{(c_{ij}+1)}\widetilde{\boldsymbol{D}}\boldsymbol{e}_{(c_{ij'}+1)}^T + \tilde{\sigma}^2 I(j=j') = a_{ij(c_{ij})}a_{ij'(c_{ij'})}\tilde{d}_{(c_{ij}),(c_{ij'})} + a_{ij(c_{ij})}a_{ij'(c_{ij'}+1)}$ $\tilde{d}_{(c_{ij}),(c_{ij'}+1)} + a_{ij(c_{ij}+1)}a_{ij'(c_{ij'})}\tilde{d}_{(c_{ij}+1),(c_{ij'})} + a_{ij(c_{ij}+1)}a_{ij'(c_{ij'}+1)}\tilde{d}_{(c_{ij}+1),(c_{ij'}+1)} + \tilde{\sigma}^2 I(j=j')$. Therefore, $\boldsymbol{p}_i\widetilde{\boldsymbol{D}}\boldsymbol{p}_i^T + \tilde{\sigma}^2\boldsymbol{I}_{n_i} = \boldsymbol{0}$ is equivalent to the system of equations, $\boldsymbol{A}_i\boldsymbol{X} = \boldsymbol{0}$, where $\boldsymbol{A}_i$ is a $[\frac{1}{2}n_i(n_i+1)] \times [\frac{1}{2}k(k+1)+1]$ matrix whose non-zero elements are $a_{ij(c_{ij})}a_{ij'(c_{ij'})}$, $a_{ij(c_{ij})}a_{ij'(c_{ij'}+1)}$, $a_{ij(c_{ij}+1)}a_{ij'(c_{ij'})}$, $a_{ij(c_{ij}+1)}a_{ij'(c_{ij'}+1)}$, and $I(j=j')$. The locations of the first four non-zero numbers depend on the values of c_{ij} and $c_{ij'}$ while $I(j=j')$ is the last element of $\boldsymbol{A}_i$. Therefore, $\boldsymbol{P}\widetilde{\boldsymbol{\mathcal{D}}}\boldsymbol{P}^T + \tilde{\sigma}^2\boldsymbol{I}_N = \boldsymbol{0}$ if and only if $\boldsymbol{p}_i\widetilde{\boldsymbol{D}}\boldsymbol{p}_i^T + \tilde{\sigma}^2\boldsymbol{I}_{n_i} = \boldsymbol{0}$ for $\forall i$, or if and only if $\boldsymbol{AX} = \boldsymbol{0}$.

Theorem 1. The proposed Bayesian mixed effects model is identifiable if and only if rank$(\boldsymbol{A}) = \frac{1}{2}k(k+1)+1$.

(Proof) The proof follows from Lemma 1. See Schott [1] for details.

Full Conditional Posterior Distributions

We here derive full conditional posteriors for all unknown parameters from the joint posterior distribution and describe the MCMC schemes.

Conditional Posterior of $\boldsymbol{\gamma}$

The full conditional posterior distribution of the indicator variable γ_a can be expressed as

$$P(\gamma_a = 1|\boldsymbol{\gamma}_{-a}, \boldsymbol{\theta}_{-\beta_a}, \boldsymbol{y}) = \frac{w_a L_{a1}}{(1-w_a)L_{a0} + w_a L_{a1}},$$

where $L_{am} = P(\boldsymbol{y}|\gamma_a = m, \boldsymbol{\gamma}_{-a}, \boldsymbol{\theta}_{-\beta_a})$ for $m = 1,0$. Suppose that the prior of β_a is $P(\beta_a|\gamma_a, \sigma_\beta^2) \overset{d}{=} N(0, \gamma_a \sigma_\beta^2)$. We first derive the joint distribution of $\boldsymbol{y}$ and β_a:

$P(\boldsymbol{y}, \beta_a|\gamma_a = 1, \boldsymbol{\gamma}_{-a}, \boldsymbol{\theta}_{-\beta_a}) \propto P(\boldsymbol{y}|\gamma_a = 1, \boldsymbol{\gamma}_{-a}, \boldsymbol{\theta})P(\beta_a|\sigma_\beta^2) \propto exp(-\frac{1}{2\sigma^2}\sum_{i=1}^{n}\sum_{j=1}^{n_i}(y_{ij} - \mu - \boldsymbol{x}_{ij}\boldsymbol{\beta} - \boldsymbol{v}_{ij}\boldsymbol{b})^2)\, exp\left(-\frac{\beta_a^2}{2\sigma_\beta^2}\right) \propto exp(-\frac{1}{2}\{\frac{\sum_{i=1}^{n}\sum_{j=1}^{n_i}(c_{ij}+x_{ija}\beta_a)^2)}{\sigma^2} - \left(\frac{\sum_{i=1}^{n}\sum_{j=1}^{n_i}x_{ija}(c_{ij}+x_{ija}\beta_a)}{\sigma^2}\right)^2 (\tilde{\sigma}_\beta^2)^{-1} + \frac{(\beta_a - \tilde{\mu}_a)^2}{\tilde{\sigma}_\beta^2}\})$, where $\tilde{\mu}_a = (\tilde{\sigma}_\beta^2)^{-1}\sum_{i=1}^{n}\sum_{j=1}^{n_i}x_{ija}\,(c_{ij} + x_{ija}\beta_a)/\sigma^2$, $\tilde{\sigma}_\beta^2 = \sigma_\beta^{-2} + \sigma^{-2}\sum_{i=1}^{n}\sum_{j=1}^{n_i}x_{ija}^2$ and $c_{ij} = y_{ij} - \mu - \boldsymbol{x}_{ij}\boldsymbol{\beta} - \boldsymbol{v}_{ij}\boldsymbol{b}$. L_{a1} and L_{a0} can be calculated as follows:

$$L_{a1} = P(\boldsymbol{y}|\gamma_a = 1, \boldsymbol{\gamma}_{-a}, \boldsymbol{\theta}_{-\beta_a}) = \int_{\beta_a} P(\boldsymbol{y}, \beta_a|\gamma_a = 1, \boldsymbol{\gamma}_{-a}, \boldsymbol{\theta}_{-\beta_a})\, d\beta_a$$

$$\propto (\tilde{\sigma}_\beta^2)^{-\frac{1}{2}} exp(-\frac{1}{2}\{\frac{\sum_{i=1}^{n}\sum_{j=1}^{n_i}(c_{ij} + x_{ija}\beta_a)^2)}{\sigma^2} - \left(\frac{\sum_{i=1}^{n}\sum_{j=1}^{n_i}x_{ija}\,(c_{ij} + x_{ija}\beta_a)}{\sigma^2}\right)^2 (\tilde{\sigma}_\beta^2)^{-1}\}),$$

$$L_{a0} \propto (\sigma_\beta^2)^{-\frac{1}{2}} exp(-\frac{1}{2}\{\frac{\sum_{i=1}^{n}\sum_{j=1}^{n_i}(c_{ij}+x_{ija}\beta_a)^2)}{\sigma^2}\}).$$

The Metropolis-Hastings scheme is employed as below. Suppose the current γ_a is c (=0 or 1) and a new value d (=0 or 1) is proposed from the prior probability $P(\gamma_a = c)$. If $c = d$, the acceptance probability for the Metropolis-Hastings scheme is set to 1, so that γ_a remains at c without need of update. Otherwise, we update γ_a from the current value c to $d = 1 - c$ with the acceptance probability as below:

$$\alpha = min\left(1, \left(\frac{1-w_a}{w_a}R\right)^{1-2c}\right) \; where\; R = \frac{L_{a1}}{L_{a0}} = \left(\frac{\tilde{\sigma}_\beta^2}{\sigma_\beta^2}\right)^{-\frac{1}{2}} exp\left(\frac{1}{2}\left(\frac{\sum_{i=1}^{n}\sum_{j=1}^{n_i}x_{ija}(c_{ij}+x_{ija}\beta_a)}{\sigma^2}\right)^2 (\tilde{\sigma}_\beta^2)^{-1}\right)$$

Conditional posterior of $\boldsymbol{\lambda}$

The full conditional posterior distribution for the ath putative marker location is

$$P(\lambda_a|\boldsymbol{\gamma}, \boldsymbol{\lambda}_{-a}, \boldsymbol{y}) = \begin{cases} P(\boldsymbol{y}|\boldsymbol{\gamma}, \boldsymbol{\theta})P(\lambda_a) & if\; \gamma_a = 1 \\ P(\lambda_a) & if\; \gamma_a = 0 \end{cases}$$

Since this conditional distribution has a nonstandard form, the Metropolis-Hastings algorithm is needed to update λ_a. First, a new location λ_a^* is sampled from $q(\lambda_a^*; \lambda_a)$ for which we can employ the uniform distribution $U[\lambda_a - d, \lambda_a + d]$ where d is a pre-determined tuning number (e.g., $d = 2$). A proposal for the new location is accepted or rejected with the acceptance probability $\alpha = min(1, \frac{P(\lambda_a^*|\boldsymbol{\gamma}, \boldsymbol{\lambda}_{-a}, \boldsymbol{y})q(\lambda_a; \lambda_a^*)}{P(\lambda_a|\boldsymbol{\gamma}, \boldsymbol{\lambda}_{-a}, \boldsymbol{y})q(\lambda_a^*; \lambda_a)})$.

Conditional posterior of $\boldsymbol{b}$

Let $\boldsymbol{\mu} = \mu\mathbf{1}_N$ and $\boldsymbol{x} = (\boldsymbol{x}_i, \dots, \boldsymbol{x}_n)^T$. The full conditional posterior distribution of the latent normal variable $\boldsymbol{b}$ is given by

$$P(\boldsymbol{b}|\boldsymbol{y},\boldsymbol{\gamma},\boldsymbol{\theta}_{-\boldsymbol{b}}) \propto exp\left\{-\frac{1}{2\sigma^2}(\boldsymbol{y}-\boldsymbol{\mu}-\boldsymbol{x\beta}-\boldsymbol{vb})^T(\boldsymbol{y}-\boldsymbol{\mu}-\boldsymbol{x\beta}-\boldsymbol{vb})\right\}exp\left(-\frac{1}{2}\boldsymbol{b}^T\boldsymbol{b}\right)$$

$$\propto exp\left\{-\frac{1}{2}(\boldsymbol{b}-\boldsymbol{b}^*)^T\left(\frac{1}{\sigma^2}\boldsymbol{v}^T\boldsymbol{v}+\boldsymbol{I}_{nk}\right)(\boldsymbol{b}-\boldsymbol{b}^*)\right\}.$$

Thus, $\boldsymbol{b}|\boldsymbol{y},\boldsymbol{\gamma},\boldsymbol{\theta}_{-\boldsymbol{b}} \sim N_{nk}(\boldsymbol{b}^*, \boldsymbol{\Sigma}_b^*)$, where $\boldsymbol{\Sigma}_b^* = \left(\frac{1}{\sigma^2}\boldsymbol{v}^T\boldsymbol{v}+\boldsymbol{I}_{nk}\right)^{-1}$ and $\boldsymbol{b}^* = \frac{1}{\sigma^2}\boldsymbol{\Sigma}_b^*\boldsymbol{v}^T((\boldsymbol{y}-\boldsymbol{\mu}-\boldsymbol{x\beta})$.

Conditional posterior of $\boldsymbol{\delta}$

In order to obtain the full conditional distribution of $\boldsymbol{\delta}$, we rewrite model 2 as

$$y_{ij} = \mu + \boldsymbol{x}_{ij}\boldsymbol{\beta} + \sum_{l=1}^{k}\delta_l(\,p_{ijl}(b_{il} + \sum_{m=1}^{l-1} b_{im}\psi_{lm})) + e_{ij},$$

and define the $k \times 1$ vector $\boldsymbol{w}_{ij} = (w_{ij1}, \dots, w_{ijk})^T = (\,p_{ijl}(b_{il} + \sum_{m=1}^{l-1} b_{im}\psi_{lm}) : l = 1, \dots, k)^T$ and $\xi_{ijl} = y_{ij} - \mu - \boldsymbol{x}_{ij}\boldsymbol{\beta} - \Sigma_{m \neq l}\, w_{ijm}\delta_m$. The full conditional distribution of $\boldsymbol{\delta}$ is given by

$$P(\boldsymbol{\delta}|\boldsymbol{y},\boldsymbol{\gamma},\boldsymbol{\theta}_{-\boldsymbol{\delta}}) \propto exp\left\{-\frac{1}{2\sigma^2}(\boldsymbol{y}-\boldsymbol{\mu}-\boldsymbol{x\beta}-\boldsymbol{w\delta})^T(\boldsymbol{y}-\boldsymbol{\mu}-\boldsymbol{x\beta}-\boldsymbol{w\delta})\right\}$$

$$\times \prod_{l=1}^{k}\left\{\frac{1}{\sqrt{2\pi}s_{l0}}exp\left(-\frac{1}{2s_{l0}^2}(\delta_l - m_{l0})^2\right)I(\delta_l > 0)\right\},$$

$$P(\delta_l|\boldsymbol{y},\boldsymbol{\gamma},\boldsymbol{\theta}_{-\delta_l}) \propto exp\left\{-\frac{1}{2\sigma^2}(\boldsymbol{\xi}_l - \boldsymbol{w}_l\delta_l)^T(\boldsymbol{\xi}_l - \boldsymbol{w}_l\delta_l)\right\}$$

$$\times\left\{\frac{1}{\sqrt{2\pi}s_{l0}}exp\left(-\frac{1}{2s_{l0}^2}(\delta_l - m_{l0})^2\right)I(\delta_l > 0)\right\}$$

$$\propto \left\{\frac{1}{\sqrt{2\pi}\sigma_l^*}exp\left(-\frac{1}{2\sigma_l^{*2}}(\delta_l - \delta_l^*)^2\right)I(\delta_l > 0)\right\}.$$

where $\boldsymbol{w} = (\boldsymbol{w}_{11}, \dots, \boldsymbol{w}_{nn_n})^T$, $\boldsymbol{w}_l = (w_{11l}, \dots, w_{nn_nl})^T$ and $\boldsymbol{\xi}_l = (\xi_{11l}, \dots, \xi_{nn_nl})^T$. That is, $\delta_l|\boldsymbol{y},\boldsymbol{\gamma},\boldsymbol{\theta}_{-\delta_l} \sim N^+(\delta_l^*, \sigma_l^{*2})$, where $\sigma_l^{*2} = \left(\frac{1}{\sigma^2}\boldsymbol{w}_l^T\boldsymbol{w}_l + s_{l0}^{-2}\right)^{-1}$ and $\delta_l^* = \sigma_l^{*2}\left(\frac{1}{\sigma^2}\boldsymbol{w}_l^T\boldsymbol{\xi}_l + s_{l0}^{-2}m_{l0}\right)$.

Conditional posterior of $\boldsymbol{\psi}$

In order to obtain the full conditional distribution of $\boldsymbol{\psi}$, we rewrite model 2 as

$$y_{ij} = \mu + \boldsymbol{x}_{ij}\boldsymbol{\beta} + \sum_{l=1}^{k} b_{il}(\delta_l p_{ijl} + \sum_{m=l+1}^{k} \delta_m p_{ijm}\psi_{ml}) + e_{ij},$$

and define the $k(k-1)/2 \times 1$ vector $\boldsymbol{u}_{ij} = \left(b_{il}\delta_m p_{ijm}: l = 1,\dots,k, m = l+1,\dots,k\right)^T$. The full conditional distribution of $\boldsymbol{\psi}$ is given by

$$P(\boldsymbol{\psi}|\boldsymbol{y},\boldsymbol{\gamma},\boldsymbol{\theta}_{-\psi}) \propto exp\left\{-\frac{1}{2\sigma^2}(\boldsymbol{y}-\boldsymbol{\mu}-\boldsymbol{x\beta}-\boldsymbol{u\psi})^T(\boldsymbol{y}-\boldsymbol{\mu}-\boldsymbol{x\beta}-\boldsymbol{u\psi})\right\}$$
$$\times\ exp\left(-\frac{1}{2}(\boldsymbol{\psi}-\boldsymbol{\psi}_0)^T\,\boldsymbol{R}_0^{-1}(\boldsymbol{\psi}-\boldsymbol{\psi}_0)\right)$$
$$\propto exp\left\{-\frac{1}{2}(\boldsymbol{\psi}-\boldsymbol{\psi}^*)^T\boldsymbol{\Sigma}_{\psi}^{*\ -\mathbf{1}}(\boldsymbol{\psi}-\boldsymbol{\psi}^*)\right\},$$

where $\boldsymbol{u} = \left(\boldsymbol{u}_{11},\dots,\boldsymbol{u}_{nn_n}\right)^T$. That is, $\boldsymbol{\psi}|\boldsymbol{y},\boldsymbol{\gamma},\boldsymbol{\theta}_{-\boldsymbol{\psi}} \sim N(\boldsymbol{\psi}^*,\boldsymbol{\Sigma}_{\psi}^*)$, where $\boldsymbol{\Sigma}_{\psi}^* = \left(\frac{1}{\sigma^2}\boldsymbol{u}^T\boldsymbol{u} + \boldsymbol{R}_0^{-1}\right)^{-1}$ and $\boldsymbol{\psi}^* = \boldsymbol{\Sigma}_{\psi}^*\left(\frac{1}{\sigma^2}\boldsymbol{u}^T(\boldsymbol{y}-\boldsymbol{\mu}-\boldsymbol{x\beta}) + \boldsymbol{R}_0^{-1}\boldsymbol{\psi}_0\right)$.

Conditional posterior of $\boldsymbol{\beta}$ and σ_β^2

Suppose that the prior distribution of β_a is $P(\beta_a|\gamma_a,\sigma_\beta^2) \overset{d}{=} N(0,\gamma_a\sigma_\beta^2)$. If $\gamma_a = 0$, $\beta_a = 0$. Otherwise, β_a is generated from its conditional posterior distribution

$$P(\beta_a|\gamma_a = 1,\boldsymbol{\gamma}_{-a},\boldsymbol{\theta}_{-\beta_a},\boldsymbol{y}) \propto P(\boldsymbol{y}|\gamma_a = 1,\boldsymbol{\gamma}_{-a},\boldsymbol{\theta})P(\beta_a|\gamma_a = 1,\sigma_\beta^2) \propto exp\left(-\frac{(\beta_a-\tilde{\mu}_a)^2}{2\tilde{\sigma}_\beta^{\ 2}}\right).$$

That is, $\beta_a|\gamma_a = 1,\boldsymbol{\gamma}_{-a},\boldsymbol{\theta}_{-\beta_a},\boldsymbol{y} \sim N(\tilde{\mu}_a,\tilde{\sigma}_\beta^{\ 2})$, where $\tilde{\mu}_a = \left(\tilde{\sigma}_\beta^{\ 2}\right)^{-1}\sum_{i=1}^{n}\sum_{j=1}^{n_i}x_{ija}\left(c_{ij}+x_{ija}\beta_a\right)/\sigma^2$, $\tilde{\sigma}_\beta^{\ 2} = \sigma_\beta^{-2} + \sigma^{-2}\sum_{i=1}^{n}\sum_{j=1}^{n_i}x_{ija}^2$ and $c_{ij} = y_{ij}-\mu-\boldsymbol{x}_{ij}\boldsymbol{\beta}-\boldsymbol{v}_{ij}\boldsymbol{b}$. The full conditional posterior distribution of hyperparameter σ_β^2 is given by

$$P(\sigma_\beta^2|\beta_a) \propto P(\beta_a|\sigma_\beta^2)P(\sigma_\beta^2) \propto (\sigma_\beta^2)^{-\frac{\nu_\beta+1}{2}-1}exp\left(-\frac{\beta_a^2+\nu_\beta s_\beta^2}{2\sigma_\beta^2}\right).$$

That is, $\sigma_\beta^2|\beta_a \sim Inv-\chi^2(\nu_\sigma + 1,(\beta_a^2+\nu_\beta s_\beta^2)/(\nu_\beta+1))$.

Conditional posterior of $\boldsymbol{\mu}$ and $\boldsymbol{\sigma^2}$

The full conditional posterior distribution for μ is given by

$$P(\mu|\boldsymbol{\gamma},\boldsymbol{\theta}_{-\mu},\boldsymbol{y}) \propto P(\boldsymbol{y}|\boldsymbol{\gamma},\boldsymbol{\theta})P(\mu) \propto exp\left(-\frac{1}{2}(\mu-\mu^*)^T\,(\frac{1}{\sigma^2}+\frac{1}{s_y^2})(\mu-\mu^*)\right).$$

That is, $\mu|\boldsymbol{\gamma},\boldsymbol{\theta}_{-\mu},\boldsymbol{y} \sim N(\mu^*,\sigma_\mu^{2*})$ where $\sigma_\mu^{2*}=\left(\frac{1}{\sigma^2}+\frac{1}{s_y^2}\right)^{-1}$, $\mu^*=\frac{1}{\sigma^2}(\boldsymbol{y}-\boldsymbol{x\beta}-\boldsymbol{vb})^T(\boldsymbol{y}-\boldsymbol{x\beta}-\boldsymbol{vb})+\frac{1}{s_y^2}\bar{y}$, $\bar{y}=\frac{1}{N}\sum_{i=1}^{n}\sum_{j=1}^{n_i}y_{ij}$ and $s_y^2=\frac{1}{N-1}\sum_{i=1}^{n}\sum_{j=1}^{n_i}(y_{ij}-\bar{y})^2$. The full conditional posterior distribution for σ^2 is given by

$$P(\sigma^2|\boldsymbol{\gamma},\boldsymbol{\theta}_{-\sigma^2},\boldsymbol{y}) \propto P(\boldsymbol{y}|\boldsymbol{\gamma},\boldsymbol{\theta})P(\sigma^2) \propto (\sigma^2)^{-\frac{\nu_\sigma+N}{2}-1}\, exp\left(-\frac{\nu_\sigma s_\sigma^2+N\hat{\sigma}^2}{2\sigma^2}\right).$$

That is, $\sigma^2|\boldsymbol{\gamma},\boldsymbol{\theta}_{-\sigma^2},\boldsymbol{y} \sim\ Inv-\chi^2(\nu_\sigma+N,\frac{\nu_\sigma s_\sigma^2+N\hat{\sigma}^2}{\nu_\sigma+N})$ where $\hat{\sigma}^2=\frac{1}{N}(\boldsymbol{y}-\boldsymbol{\mu}-\boldsymbol{x\beta}-\boldsymbol{vb})^T(\boldsymbol{y}-\boldsymbol{\mu}-\boldsymbol{x\beta}-\boldsymbol{vb})$.

Reference

1. Schot JR. Matrix Analysis for Statistics. Hoboken: John Wiley & Sons, 2016.
