# Supplementary material for: Bayesian mixed models for longitudinal genetic data: theory, concepts, and simulation studies
Source: Genomics Inform. 2022 Mar 31;20(1):e8. doi: 10.5808/gi.21080 (PMC9001998; doi:10.5808/gi.21080)

Supplementary Fig. 1. Time-dependent curves of averaged phenotype values for three different genotypes (0, 1, 2) at 1st causal single nucleotide polymorphism (SNP) with no SNP-time interaction and 10th causal SNP with SNP-time interaction for Setups 4 and 5.

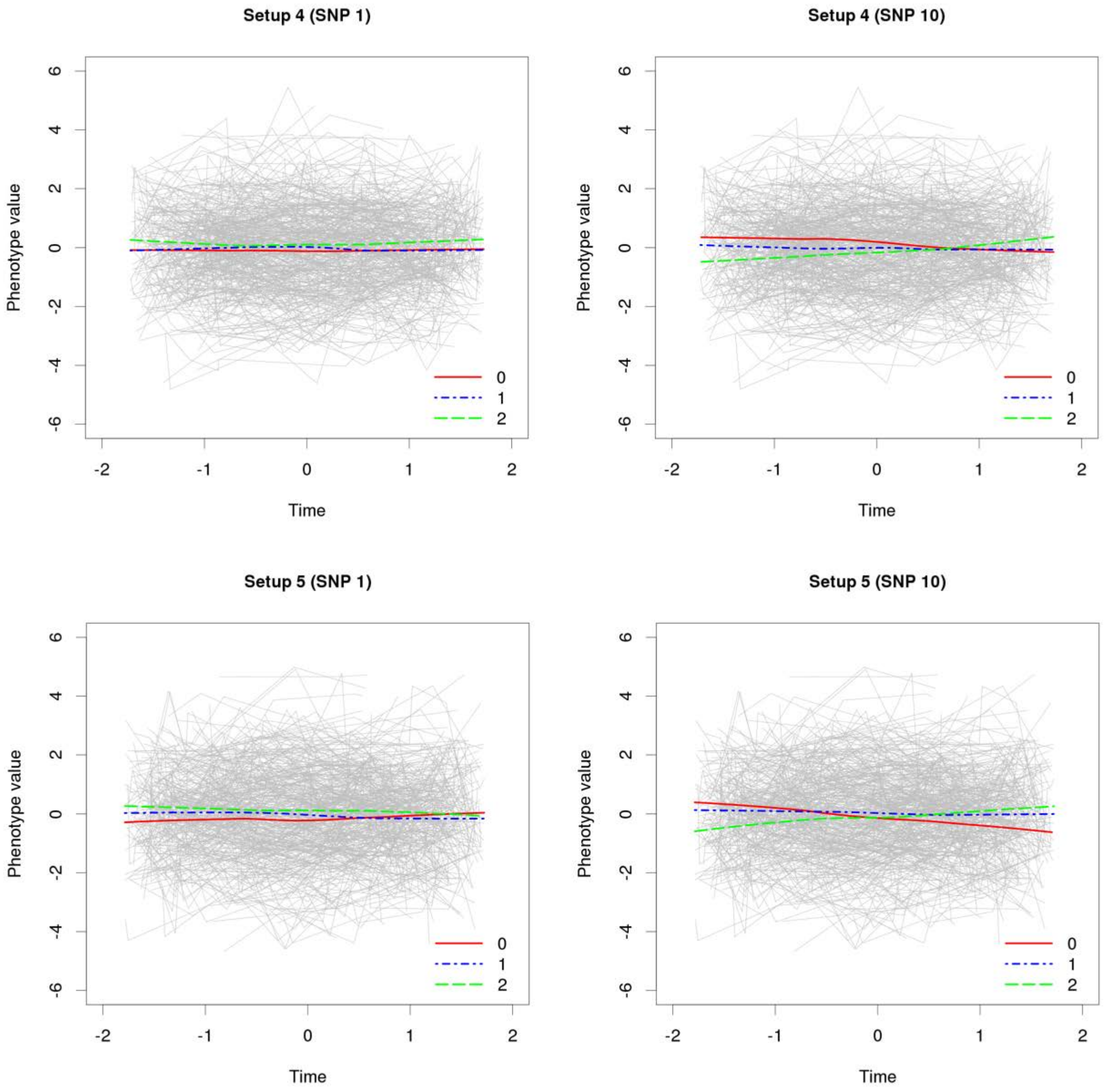

Supplement: Supplementary Fig. 1. — Time-dependent curves of averaged phenotype values for three different genotypes (0, 1, 2) at 1st causal single nucleotide polymorphism (SNP) with no SNP-time interaction and 10th causal SNP with SNP-time interaction for Setups 4 and 5. [file gi-21080suppl2.pdf]
