# Supplementary material for: Bayesian mixed models for longitudinal genetic data: theory, concepts, and simulation studies
Source: Genomics Inform. 2022 Mar 31;20(1):e8. doi: 10.5808/gi.21080 (PMC9001998; doi:10.5808/gi.21080)

Supplementary Fig. 2. Trace plots of $\sigma^2, \delta_1, \delta_2, \delta_3, \psi_{21}, \psi_{31}\ and\ \psi_{22}$ for Setups 1–6 in the simulation study.The black lines represent the values of the draws for all parameters at each iteration and gray lines represent the true values of the parameters.

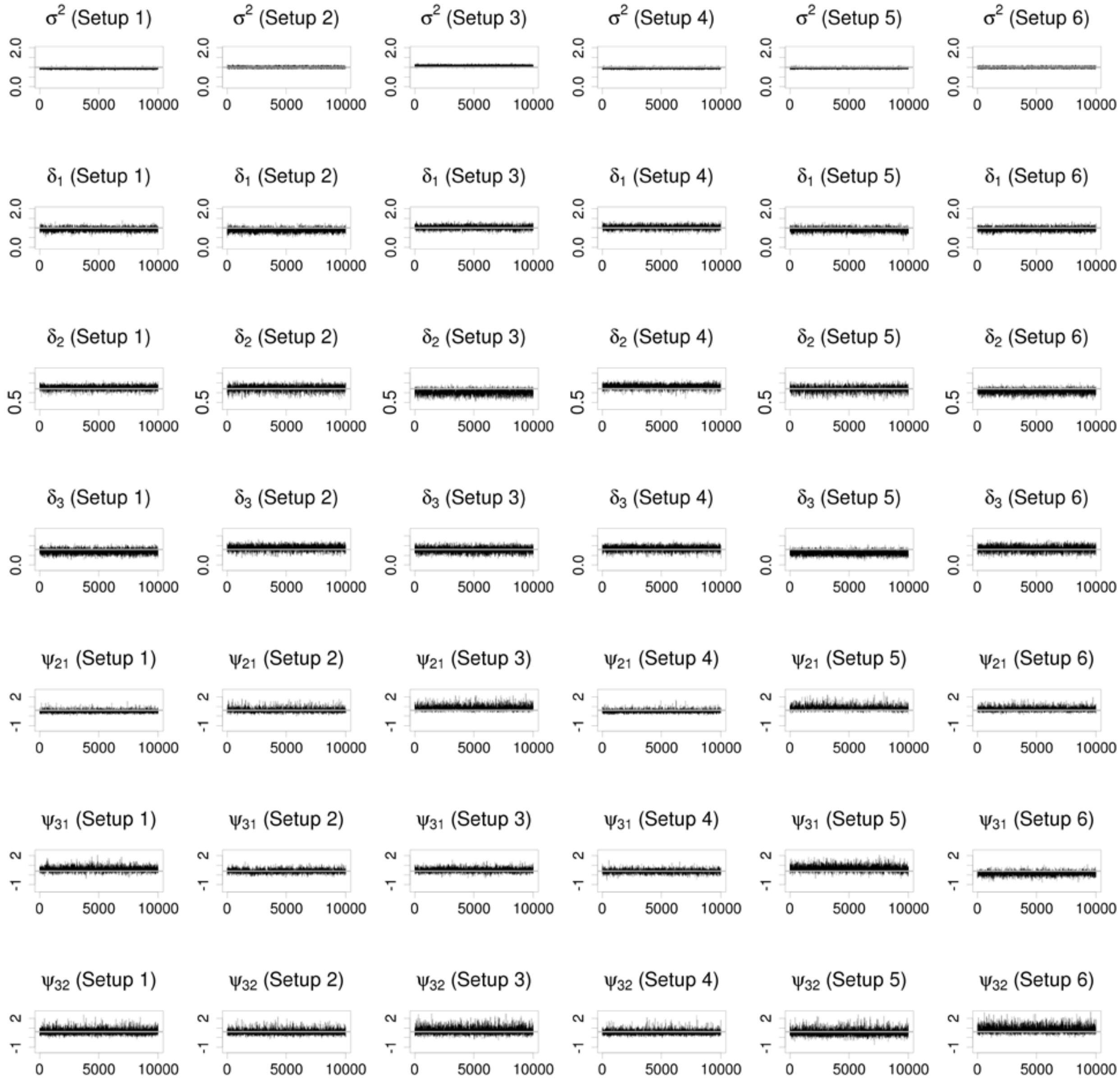

Supplement: Supplementary Fig. 2. — Trace plots of σ2, δ1, δ2, δ3, ψ21, ψ31 and ψ22 for Setups 1‒6 in the simulation study.The black lines represent the values of the draws for all parameters at each iteration and gray lines represent the true values of the parameters. [file gi-21080suppl3.pdf]
