# Supplementary material for: Bayesian mixed models for longitudinal genetic data: theory, concepts, and simulation studies
Source: Genomics Inform. 2022 Mar 31;20(1):e8. doi: 10.5808/gi.21080 (PMC9001998; doi:10.5808/gi.21080)

Supplementary Fig. 3. Posterior (solid line) and prior (dashed line) densities of the parameters for random errors and random effects for Setups 1–6. Estimated densities are based on 10,000 random draws.

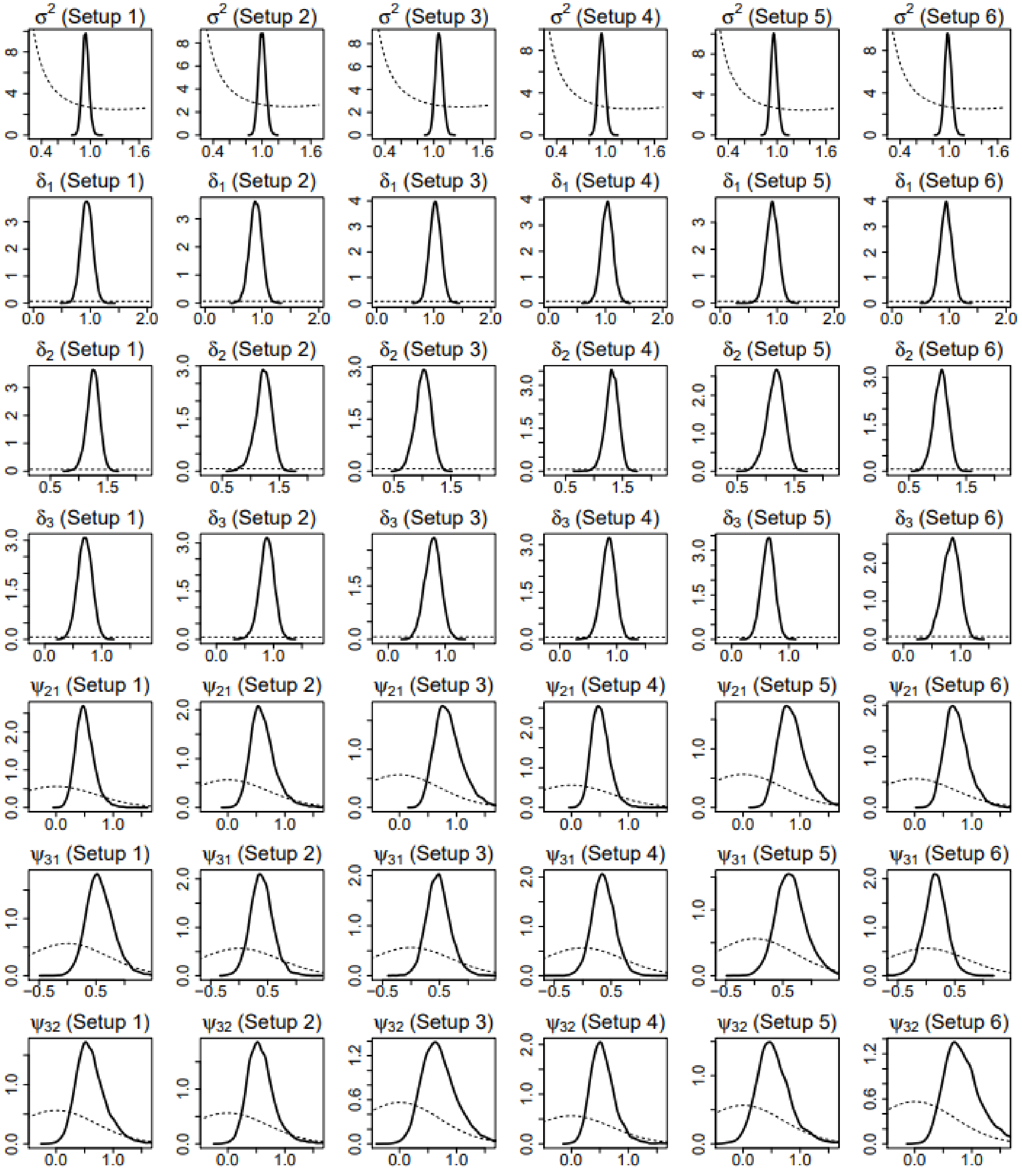

Supplement: Supplementary Fig. 3. — Posterior (solid line) and prior (dashed line) densities of the parameters for random errors and random effects for Setups 1‒6. Estimated densities are based on 10, 000 random draws. [file gi-21080suppl4.pdf]
