# Supplementary material for: Bayesian mixed models for longitudinal genetic data: theory, concepts, and simulation studies
Source: Genomics Inform. 2022 Mar 31;20(1):e8. doi: 10.5808/gi.21080 (PMC9001998; doi:10.5808/gi.21080)

Supplementary Fig. 4. 95% highest posterior density (HPD) intervals for $\sigma^2, \delta_1, \delta_2, \delta_3, \psi_{21}, \psi_{31}$ *and* ψ_{22} for Setups 1–6. The blue lines represent the 95% HPD intervals (100 replicates).

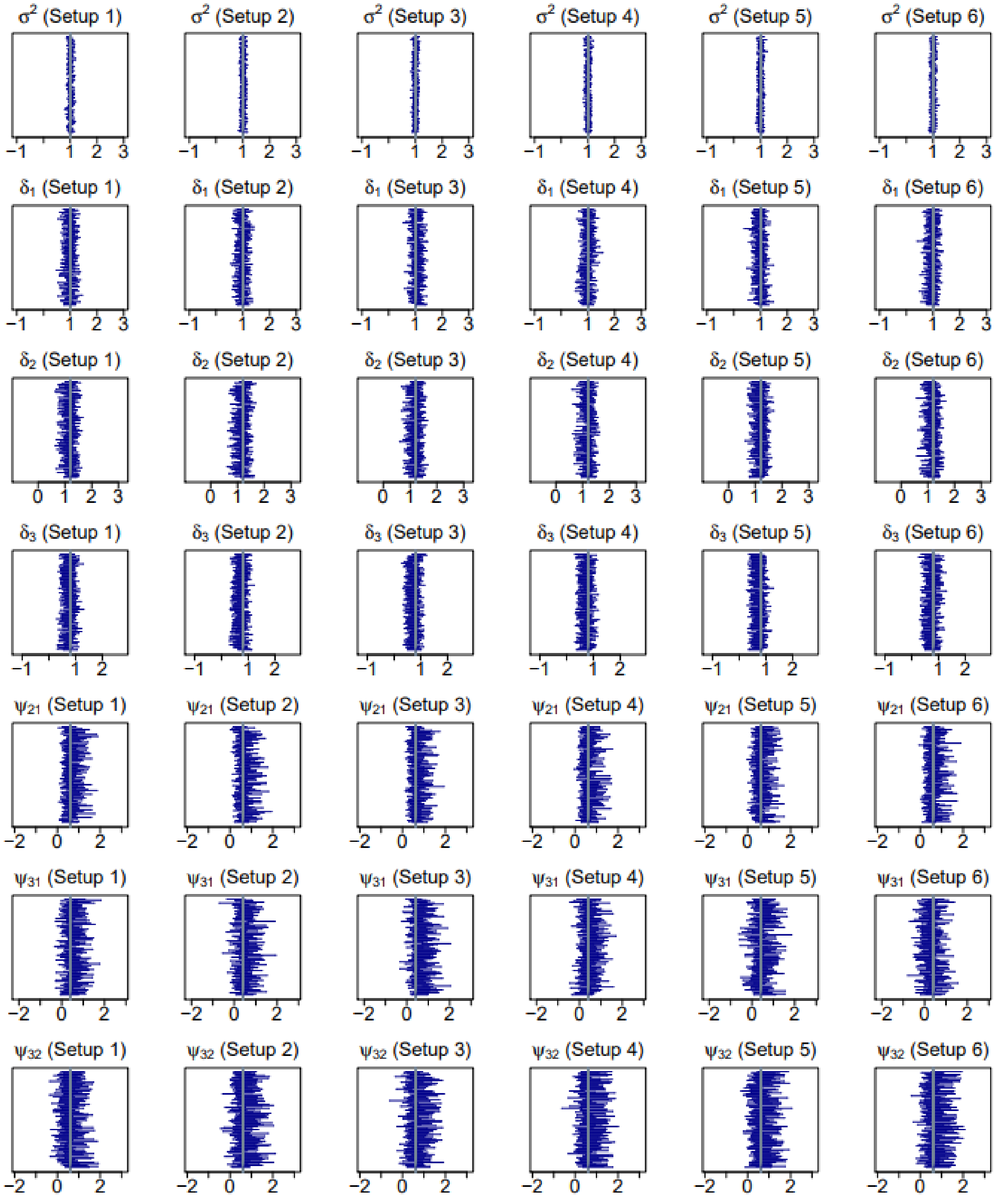

Supplement: Supplementary Fig. 4. — 95% highest posterior density (HPD) intervals for σ2, δ1, δ2, δ3, ψ21, ψ31 and ψ22 for Setups 1‒6. The blue lines represent the 95% HPD intervals (100 replicates). [file gi-21080suppl5.pdf]
