# Supplementary material for: Bayesian mixed models for longitudinal genetic data: theory, concepts, and simulation studies
Source: Genomics Inform. 2022 Mar 31;20(1):e8. doi: 10.5808/gi.21080 (PMC9001998; doi:10.5808/gi.21080)
Supplement: Supplementary Table 1. — Posterior means, medians, standard deviations and 95% HPD intervals of the parameters for random errors and random effects in the simulation study for sample size [file gi-21080suppl6.pdf]

Supplementary Table 1. Posterior means, medians, standard deviations and 95% HPD intervals of the parameters for random errors and random effects in the simulation study for sample size

# Sample	Par	True	Mean	Med	SD	95% HPD
100	σ^2	1	0.97	0.96	0.12	0.75 to 1.23
	δ_1	1	0.78	0.78	0.43	0.05 to 1.59
	δ_2	1.2	1.17	1.18	0.34	(0.48 to 1.82)
	δ_3	0.8	0.56	0.55	0.30	(0.04 to 1.17)
	ψ_{21}	0.6	0.49	0.50	0.56	(-0.69 to 1.58)
	ψ_{31}	0.4	0.54	0.58	0.65	(-0.86 to 1.73)
	ψ_{32}	0.6	0.39	0.40	0.56	(-0.76 to 1.50)
200	σ^2	1	0.99	0.99	0.08	0.83 to 1.17
	δ_1	1	1.17	1.17	0.25	0.66 to 1.64
	δ_2	1.2	1.23	1.23	0.23	0.77 to 1.69
	δ_3	0.8	0.76	0.75	0.20	0.39 to 1.17
	ψ_{21}	0.6	0.83	0.79	0.37	0.22 to 1.66
	ψ_{31}	0.4	1.01	0.99	0.43	0.21 to 1.90
	ψ_{32}	0.6	0.53	0.51	0.42	–0.25 to 1.44
300	σ^2	1	1.00	0.99	0.07	0.87 to 1.14
	δ_1	1	1.19	1.19	0.20	0.80 to 1.58
	δ_2	1.2	1.20	1.21	0.21	0.78 to 1.61
	δ_3	0.8	0.79	0.79	0.17	0.47 to 1.13
	ψ_{21}	0.6	0.89	0.85	0.32	0.38 to 1.63
	ψ_{31}	0.4	0.94	0.90	0.36	0.32 to 1.74
	ψ_{32}	0.6	0.58	0.56	0.36	–0.05 to 1.37
400	σ^2	1	1.00	0.99	0.06	0.88 to 1.12
	δ_1	1	1.18	1.18	0.16	0.88 to 1.50
	δ_2	1.2	1.22	1.23	0.18	0.85 to 1.57
	δ_3	0.8	0.79	0.78	0.15	0.50 to 1.09
	ψ_{21}	0.6	0.82	0.78	0.28	0.37 to 1.46
	ψ_{31}	0.4	0.91	0.88	0.31	0.38 to 1.61
	ψ_{32}	0.6	0.61	0.58	0.34	0.00 to 1.36

Par, parameters; True, true values of parameters; SD, standard deviation; HPD, highest posterior density.
