# Supplementary material for: Bayesian mixed models for longitudinal genetic data: theory, concepts, and simulation studies
Source: Genomics Inform. 2022 Mar 31;20(1):e8. doi: 10.5808/gi.21080 (PMC9001998; doi:10.5808/gi.21080)
Supplement: Supplementary Table 2. — Posterior means, medians, standard deviations, and 95% HPD intervals of the parameters for random errors and random effects in the simulation study for number of SNPs [file gi-21080suppl7.pdf]

Supplementary Table 2. Posterior means, medians, standard deviations, and 95% HPD intervals of the parameters for random errors and random effects in the simulation study for number of SNPs

# SNP	Par	True	Mean	Med	SD	95% HPD
1000	σ^2	1	1.00	0.99	0.06	0.88 to 1.12
	δ_1	1	1.18	1.18	0.16	0.88 to 1.50
	δ_2	1.2	1.22	1.23	0.18	0.85 to 1.57
	δ_3	0.8	0.79	0.78	0.15	0.50 to 1.09
	ψ_{21}	0.6	0.82	0.78	0.28	0.37 to 1.46
	ψ_{31}	0.4	0.91	0.88	0.31	0.38 to 1.61
	ψ_{32}	0.6	0.61	0.58	0.34	0.00 to 1.36
2000	σ^2	1	0.99	0.99	0.06	0.88 to 1.12
	δ_1	1.2	1.24	1.24	0.17	0.91 to 1.56
	δ_2	0.8	1.21	1.21	0.18	0.84 to 1.56
	δ_3	0.6	0.74	0.73	0.15	0.47 to 1.05
	ψ_{21}	0.4	0.94	0.90	0.30	0.45 to 1.61
	ψ_{31}	0.6	1.20	1.17	0.37	0.58 to 2.03
	ψ_{32}	0.6	0.55	0.54	0.35	–0.11 to 1.29
3000	σ^2	1	0.98	0.98	0.06	0.87 to 1.11
	δ_1	1	1.22	1.22	0.16	0.90 to 1.55
	δ_2	1.2	1.18	1.18	0.19	0.80 to 1.54
	δ_3	0.8	0.67	0.66	0.14	0.41 to 0.97
	ψ_{21}	0.6	0.98	0.94	0.31	0.47 to 1.70
	ψ_{31}	0.4	1.39	1.36	0.38	0.72 to 2.22
	ψ_{32}	0.6	0.49	0.48	0.35	–0.18 to 1.23
5000	σ^2	1	0.97	0.97	0.06	0.86 to 1.09
	δ_1	1	1.01	1.02	0.20	0.59 to 1.39
	δ_2	1.2	1.13	1.13	0.20	0.74 to 1.51
	δ_3	0.8	0.56	0.55	0.13	0.32 to 0.84
	ψ_{21}	0.6	0.96	0.92	0.35	0.37 to 1.76
	ψ_{31}	0.4	1.45	1.43	0.42	0.68 to 2.33
	ψ_{32}	0.6	0.41	0.40	0.39	–0.33 to 1.22

HPD, highest posterior density; SNP, single nucleotide polymorphism; Par, parameters; True, true values of parameters; SD, standard deviation.
