# Supplementary material for: Bayesian mixed models for longitudinal genetic data: theory, concepts, and simulation studies
Source: Genomics Inform. 2022 Mar 31;20(1):e8. doi: 10.5808/gi.21080 (PMC9001998; doi:10.5808/gi.21080)
Supplement: Supplementary Table 3. — Posterior means, medians, standard deviations, and 95% HPD intervals of the parameters for random errors and random effects in the simulation study for proportion of causal SNPs [file gi-21080suppl8.pdf]

Supplementary Table 3. Posterior means, medians, standard deviations, and 95% HPD intervals of the parameters for random errors and random effects in the simulation study for proportion of causal SNPs

% Causal	Par	True	Mean	Med	SD	95% HPD
1	σ^2	1	1.00	0.99	0.06	0.8 to 1.12
	δ_1	1	1.18	1.18	0.16	0.8 to 1.50
	δ_2	1.2	1.22	1.23	0.18	0.8 to 1.57
	δ_3	0.8	0.79	0.78	0.15	0.5 to 1.09
	ψ_{21}	0.6	0.82	0.78	0.28	0.3 to 1.46
	ψ_{31}	0.4	0.91	0.88	0.31	0.3 to 1.61
	ψ_{32}	0.6	0.61	0.58	0.34	0.0 to 1.36
2	σ^2	1	0.99	0.99	0.06	0.8 to 1.12
	δ_1	1	1.22	1.21	0.17	0.8 to 1.54
	δ_2	1.2	1.21	1.21	0.18	0.8 to 1.56
	δ_3	0.8	0.75	0.75	0.15	0.4 to 1.06
	ψ_{21}	0.6	0.89	0.86	0.30	0.4 to 1.57
	ψ_{31}	0.4	1.11	1.08	0.34	0.5 to 1.88
	ψ_{32}	0.6	0.57	0.55	0.34	–0.0 to 1.30
3	σ^2	1	0.99	0.99	0.06	0.8 to 1.11
	δ_1	1	1.20	1.20	0.17	0.8 to 1.52
	δ_2	1.2	1.19	1.19	0.19	0.8 to 1.55
	δ_3	0.8	0.71	0.70	0.15	0.4 to 1.02
	ψ_{21}	0.6	0.93	0.89	0.31	0.4 to 1.64
	ψ_{31}	0.4	1.25	1.21	0.36	0.6 to 2.05
	ψ_{32}	0.6	0.52	0.51	0.36	–0.1 to 1.28
5	σ^2	1	0.98	0.98	0.06	0.8 to 1.10
	δ_1	1	1.08	1.09	0.18	0.7 to 1.42
	δ_2	1.2	1.16	1.16	0.20	0.7 to 1.54
	δ_3	0.8	0.62	0.61	0.14	0.3 to 0.92
	ψ_{21}	0.6	0.93	0.89	0.34	0.3 to 1.70
	ψ_{31}	0.4	1.36	1.33	0.40	0.6 to 2.22
	ψ_{32}	0.6	0.47	0.45	0.39	–0.2 to 1.27

HPD, highest posterior density; SNP, single nucleotide polymorphism; Par, parameters; True, true values of parameters; SD, standard deviation.
