# Supplementary material for: Bayesian mixed models for longitudinal genetic data: theory, concepts, and simulation studies
Source: Genomics Inform. 2022 Mar 31;20(1):e8. doi: 10.5808/gi.21080 (PMC9001998; doi:10.5808/gi.21080)
Supplement: Supplementary Table 4. — Posterior means, medians, standard deviations, and 95% HPD intervals of the parameters for random errors and random effects in the simulation study for heritability [file gi-21080suppl9.pdf]

Supplementary Table 4. Posterior means, medians, standard deviations, and 95% HPD intervals of the parameters for random errors and random effects in the simulation study for heritability

h^2	Par	True	Mean	Med	SD	95% HPD
0.1	σ^2	1	1.00	1.00	0.06	0.88 to 1.12
	δ_1	1	1.00	1.00	0.15	0.78 to 1.37
	δ_2	1.2	1.19	1.20	0.18	0.83 to 1.54
	δ_3	0.8	0.71	0.71	0.16	0.45 to 1.05
	ψ_{21}	0.6	0.67	0.63	0.28	0.28 to 1.37
	ψ_{31}	0.4	0.69	0.65	0.33	0.23 to 1.50
	ψ_{32}	0.6	0.61	0.58	0.37	–0.02 to 1.41
0.20	σ^2	1	1.00	1.00	0.06	0.88 to 1.13
	δ_1	1	1.07	1.06	0.15	0.71 to 1.29
	δ_2	1.2	1.20	1.21	0.18	0.82 to 1.53
	δ_3	0.8	0.74	0.74	0.16	0.42 to 1.03
	ψ_{21}	0.6	0.72	0.69	0.28	0.23 to 1.32
	ψ_{31}	0.4	0.77	0.74	0.32	0.13 to 1.43
	ψ_{32}	0.6	0.61	0.58	0.36	–0.04 to 1.44
0.30	σ^2	1	1.00	0.99	0.06	0.88 to 1.12
	δ_1	1	1.12	1.12	0.15	0.83 to 1.43
	δ_2	1.2	1.21	1.22	0.18	0.84 to 1.56
	δ_3	0.8	0.76	0.76	0.16	0.47 to 1.07
	ψ_{21}	0.6	0.77	0.73	0.27	0.33 to 1.41
	ψ_{31}	0.4	0.84	0.81	0.31	0.31 to 1.55
	ψ_{32}	0.6	0.61	0.58	0.35	–0.01 to 1.38
0.40	σ^2	1	1.00	0.99	0.06	0.88 to 1.12
	δ_1	1	1.18	1.18	0.16	0.88 to 1.50
	δ_2	1.2	1.22	1.23	0.18	0.85 to 1.57
	δ_3	0.8	0.79	0.78	0.15	0.50 to 1.09
	ψ_{21}	0.6	0.82	0.78	0.28	0.37 to 1.46
	ψ_{31}	0.4	0.91	0.88	0.31	0.38 to 1.61
	ψ_{32}	0.6	0.61	0.58	0.34	0.00 to 1.36

HPD, highest posterior density; SNP, single nucleotide polymorphism; Par, parameters; True, true values of parameters; SD, standard deviation.
