# Supplementary material for: Bayesian mixed models for longitudinal genetic data: theory, concepts, and simulation studies
Source: Genomics Inform. 2022 Mar 31;20(1):e8. doi: 10.5808/gi.21080 (PMC9001998; doi:10.5808/gi.21080)
Supplement: Supplementary Table 5. — Average DIC scores and simplified BPIC scores over 100 replications in the simulation study for sample size, number of SNPs, proportion of causal SNPs and heritability [file gi-21080suppl10.pdf]

Supplementary Table 5. Average DIC scores and simplified BPIC scores over 100 replications in the simulation study for sample size, number of SNPs, proportion of causal SNPs and heritability

# Sample	Avg DIC	Avg Sim BPIC	# SNP	Avg DIC	Avg Sim BPIC
100	1,763.80	1,686.58	1000	6,578.43	6,654.97
200	3,429.21	3,395.12	2000	6,757.20	6,754.54
300	4,981.30	5,018.65	3000	6,866.37	6,792.60
400	6,578.43	6,654.97	5000	7,114.50	6,853.29
% Causal	Avg DIC	Avg Sim BPIC	h^2	Avg DIC	Avg Sim BPIC
1	6,578.426	6,654.97	0.1	6,510.98	6,564.37
2	6,687.484	6,713.82	0.2	6,595.90	6,628.10
3	6,787.85	6,754.39	0.3	6,602.50	6,648.34
5	7,030.012	6,836.91	0.4	6,578.43	6,654.97

Avg DIC, averaged DIC scores over 100 replications; Avg Sim BPIC, averaged simplifed BPIC scores over 100 replications.
